# Functional analysis of a bitter gustatory receptor highly expressed in the larval maxillary galea of *Helicoverpa armigera*

**DOI:** 10.1371/journal.pgen.1010455

**Published:** 2022-10-07

**Authors:** Yan Chen, Pei-Chao Wang, Shuai-Shuai Zhang, Jun Yang, Guo-Cheng Li, Ling-Qiao Huang, Chen-Zhu Wang

**Affiliations:** 1 State Key Laboratory of Integrated Management of Pest Insects and Rodents, Institute of *Z*oology, Chinese Academy of Sciences, Beijing, P. R. China; 2 CAS Center for Excellence in Biotic Interactions, University of Chinese Academy of Sciences, Beijing, P. R. China; University of Kentucky, UNITED STATES

## Abstract

Many plant secondary substances are feeding deterrents for insects and play a key role in the selection of host plants. The taste sensilla of phytophagous insects contain gustatory sensory neurons sensitive to deterrents but the molecular basis of deterrent chemoreception remains unknown. We investigated the function of Gr180, the most highly expressed bitter gustatory receptor in the maxillary galea of *Helicoverpa armigera* larvae. Functional analyses using the *Xenopus* oocyte expression system and two-electrode voltage clamp revealed that the oocytes expressing Gr180 responded to coumarin. Tip recording results showed that the medial sensilla styloconica of the maxilla of fifth instar larvae exhibited electrophysiological responses to coumarin. Two-choice feeding bioassays confirmed that coumarin inhibited larval feeding. A homozygous mutant strain of *H*. *armigera* with truncated Gr180 proteins (*Gr180*^*−/−*^) was established using the CRISPR-Cas9 system. The responses of the medial sensilla styloconica in *Gr180*^*−/−*^ to coumarin were almost abolished, and the responses to sinigrin and strychnine were also significantly decreased. Knockout of *Gr180* alleviated the feeding deterrent effects of coumarin, sinigrin, and strychnine. Thus, we conclude that Gr180 is a receptor responding to coumarin,and also participates in sensing sinigrin and strychnine. These results enhance our understanding of the gustatory sensing mechanisms of phytophagous insects to deterrents.

## Author summary

Phytophagous insects avoid eating most plants because of the presence of distasteful secondary compounds, which introduces the question of how these compounds are perceived by insects. The cotton bollworm is a very serious crop pest in the Old World, and its caterpillars mainly feed on the reproductive organs of many host plants. Here, we study the function of a gustatory receptor, Gr180, highly expressed in the taste organ of *Helicoverpa armigera* caterpillars. The oocytes of frogs expressing Gr180 selectively responded to coumarin, a bitter compound widely exist in plants. When this gene was knocked out, the response of the deterrent cells in the medial styloconic sensilla to coumarin disappeared, and the responses to other two deterrents sinigrin and strychnine were also attenuated. However, the inhibitory effect of coumarin on the sucrose cells in the lateral styloconic sensilla was not affected. At the same time, the aversive feeding responses to these compounds were reduced in the knockout caterpillars. These findings are important for revealing the mechanisms of host selection in herbivorous insects and the use of feeding deterrents for pest control.

## Introduction

How insects choose host plants is an important issue in the field of insect–plant interactions. Primary and secondary plant metabolites play a key role in host plant selection by phytophagous insects [1]. Primary plant metabolites, like sugars, sugar alcohols, and amino acids, are ubiquitous in plants and are often used as feeding stimulants by insects. Secondary plant metabolites, such as alkaloids, flavonoids, terpenoids, and phenolic compounds, are restricted to, or have much higher concentrations in certain plant taxa. Most of these compounds inhibit feeding of herbivorous insects (feeding deterrents or bitter compounds), except some are used as stimuli by specialist insects [1,2].

Herbivorous insects use taste organs to perceive plant-derived stimulants and deterrents. The mouthparts, antennae, tarsi, and ovipositors of insects are the main taste organs. Variable quantities of taste sensilla are distributed across these organs [3,4]. Two pairs of sensilla styloconica (lateral and medial sensilla styloconica) in the larval maxillary galeae of lepidopteran insects play a crucial role in larval feeding preferences [1,5,6]. Each sensillum styloconicum contains four gustatory sensory neurons (GSNs), two of which usually respond to sugars or deterrents and are referred to as the sugar cell and deterrent cell, respectively [5]. The response profiles of deterrent cells in the same sensilla vary among species. The deterrent cell of the medial sensilla styloconica of *Bombyx mori* larvae exhibit responses to strychnine nitrate, salicin, nicotine, caffeine, phloridzin, and coumarin [7,8]. The same cell in *Pieris brassicae* larvae is sensitive to Margosan-0, toosendanin, salannin, azadirachtin, and strychnine [9,10]. The response profiles of deterrent cells may also differ between the two sensilla of the same species. The deterrent cell of the lateral sensilla styloconica of *Manduca sexta* larvae responds to caffeine, salicin, and aristolochic acid, while the deterrent cell of the medial sensilla styloconica respond to aristolochic acid [11,12]. However, the molecular basis of the response spectrum of these deterrent cells is still unclear.

Gustatory receptors (GRs) expressed on the dendrites of GSNs determine the response characteristics of GSNs, including bitter, sweet and amino acid taste [3,13]. Insect GRs were first identified in *Drosophila melanogaster* [14]. Bitter receptors are the most abundant and variable clade and are assumed to respond to bitter compounds [15–17]. Functional studies of insect bitter GRs have mainly focused on *Drosophila*. A total of 33 bitter GRs have been identified on the labellum, the main taste organ of *Drosophila*. Six bitter receptors, DmGr32a, DmGr33a, Gr39a, DmGr66a, DmGr93a, and DmGr89a, commonly expressed in each of the S-type and I-type sensilla on the labellum, are considered to be the core taste receptors in response to bitter compounds [18,19]. Five of these (DmGr32a, DmGr33a, Gr39a, DmGr66a, and DmGr93a) are involved in the perception of many bitter compounds. Their knockout was observed to reduce or abolish the electrophysiological responses of 11, 16, 5, 17, and 5 bitter compounds, respectively [20]. A variable number of GRs form the heteromeric complex that detects bitter compounds in *Drosophila*. Three bitter receptors (DmGr8a, DmGr66a, and DmGr98b) are sufficient to detect L-canavanine [21]. Three gustatory receptors, DmGR33a, DmGR66a, and GR93a are functioned together in coumarin detection by the proboscis. However, GR33a, but not GR66a and GR93a, was required to avoid coumarin during oviposition [22]. It was recently found that co-expression of DmGR39a, DmGR33a, DmGR66a, and GR93a conferred several bitter responses including coumarin to a sugar neuron [20]. Five bitter receptors (DmGr22e, DmGr47a, DmGr32a, DmGr33a, and DmGr66a) are necessary but not sufficient to detect strychnine [23]. Perception of bitter compounds in *Drosophila* requires a synergistic effect of variable numbers of bitter receptors.

In recent years, bitter GRs of many lepidopteran insects have been identified using genome and transcriptome sequencing [24–29]. However, the functions of only a few of these have been reported. In *Papilio xuthus*, the bitter receptor PxutGr1 expressed in female tarsi tuned to synephrine, an oviposition stimulant, by a combination of Sf9 cell expression systems and RNA interference [30]. In *B*. *mori*, each of three putative bitter GRs heterologously expressed in HEK293T cells responded to structurally different feeding deterrents: BmGr16 and BmGr53 responded to coumarin and caffeine, and BmGr18 to coumarin, caffeine, and pilocarpine [31]. In *Plutella xylostella*, PxylGr34 expressed in the heads of larvae and adult antenna, was tuned to the feeding and oviposition deterrents brassinolide and 24-epibrassinolide [32]. In *Pieris rapae*, PrapGr28 expressed in larval and adult taste sensilla, was tuned to the feeding stimulant sinigrin [33]. These studies demonstrate that a single bitter GR can have its own tuning profile in lepidopteran insects.

*Helicoverpa armigera* is a typical polyphagous agricultural pest, which feeds on more than 300 plants in 68 families [29]. The lateral sensilla styloconica of the larval maxilla are sensitive to sucrose and azadirachtin, and the medial sensilla styloconica respond to inositol and many deterrents, including nicotine tartrate, sinigrin, rutin, salicin, strychnine, and strophanthin-K [34–36]. The gustatory neuron axons from these two sensilla project to the suboesophageal ganglion through the ipsilateral maxillary nerve and further to the brain through the ipsilateral circumoesophageal connective, ultimately determining insect behavior [37]. A total of 180 putative bitter receptors have been identified in the *H*. *armigera* genome but, to date, only HarmGr195 is known to be responsive to proline in Sf9 cells [26].

In this study, we functionally analyzed a bitter GR that is highly expressed in the larval maxillary galea of *H*. *armigera*. First, we identified the most highly expressed bitter receptor gene, *Gr180*, in the maxillary galea of *H*. *armigera* larvae via transcriptome sequencing. Second, we discovered that Gr180 is responsive to coumarin using the *Xenopus* oocyte expression system and two-electrode voltage clamp. Third, we established the homozygous mutant strain of *Gr180* using clustered regularly interspaced short palindromic repeats—associated protein-9 nuclease (CRISPR-Cas9) system. Using electrophysiological and behavioral experiments, we demonstrated that Gr180 is not only responsible for detecting coumarin but is also involved in sensing sinigrin and strychnine.

## Results

### *Gr180* was the most highly expressed GR gene in the larval maxillary galea of *H*. *armigera*

**Fig 1:**
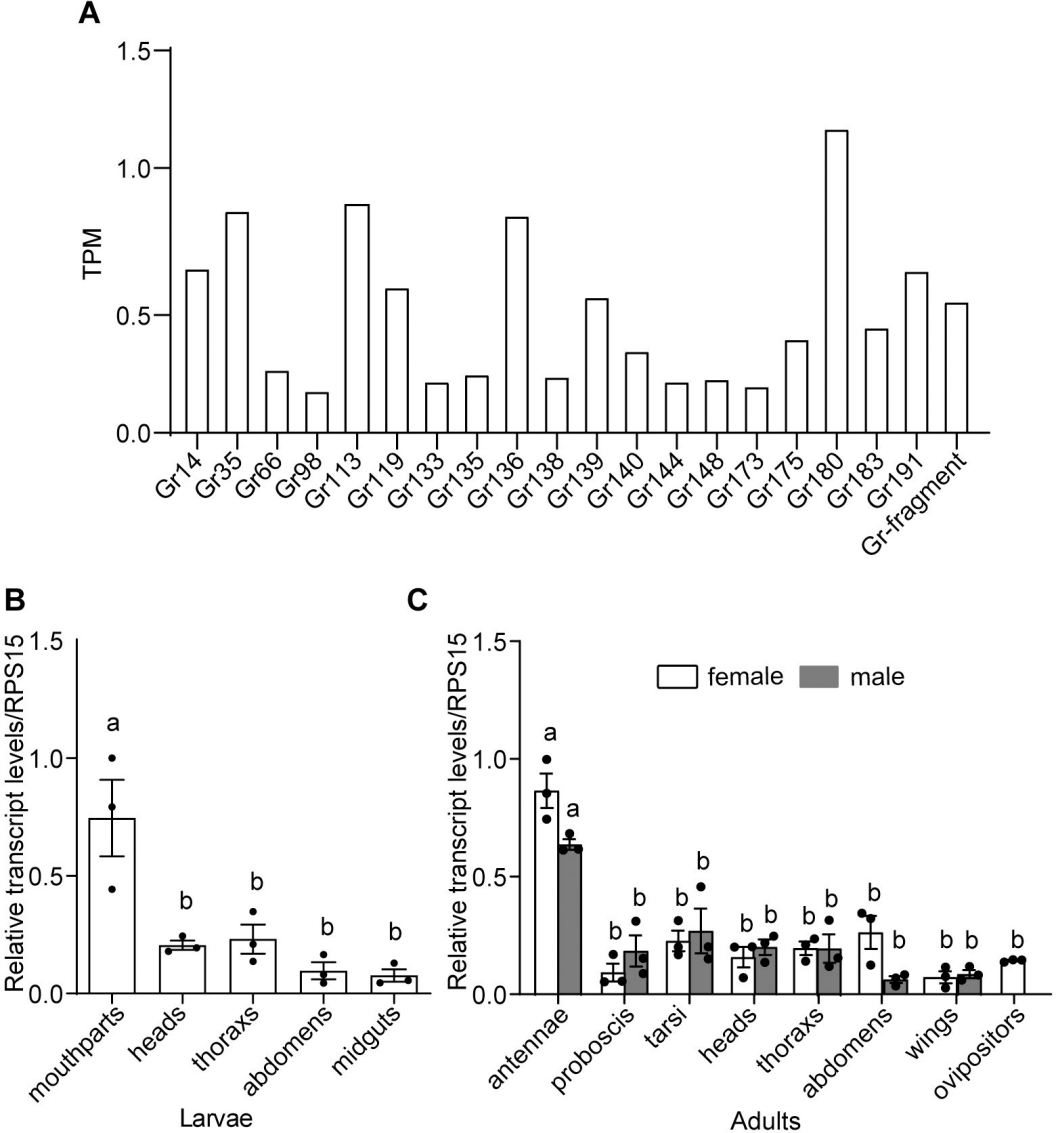
Expression patterns of gustatory receptors (GRs) in *Helicoverpa armigera*. (**A**) The TPM value of the top 20 highly expressed GRs in the larval maxillary galea of *H*. *armigera* via transcriptome sequencing. (**B**) Relative transcript levels of *Gr180* in the organs of the fifth instar larvae by qRT-PCR. (**C**) Relative transcript levels of *Gr180* in the organs of virgin female and male adults. Data are mean ± SEM, n = 3. Columns with different letters are significantly different at p < 0.05 (one-way ANOVA followed by post-hoc analysis with Tukey’s HSD test).

A total of 63 GRs were identified in the larval maxillary galea by transcriptome sequencing (S1 Table). Fig 1A lists the top 20 GRs in terms of the transcripts per kilobase of exon model per million mapped reads values (TPM); *Gr180* was the most highly expressed (Fig 1A). Since the number of GR genes of *H*. *armigera* is much more than that of *B*. *mori*, there is low levels of potential orthology between GRs of the two species, but *Gr180* has an orthologous gene, *BmGR63*, in *B*. *mori* (S1 Fig). We further examined the relative transcript levels of *Gr180* in different organs of fifth instar larvae and adults using quantitative real-time PCR. The results showed that this gene was expressed in all organs tested, with the highest expression in the mouthparts of larvae and antennae of adults (Fig 1B and 1C).

### Oocytes expressing Gr180 exhibited strong responses to coumarin

A *Xenopus* oocyte expression system with two-electrode voltage-clamp recording was used to characterize the function of Gr180. A panel of 25 phytochemicals belonging to alkaloids, flavonoids, terpenoids, glycosides, phenols, phytohormones, amino acids, and a sugar alcohol were used to screen the ligands of Gr180.

**Fig 2:**
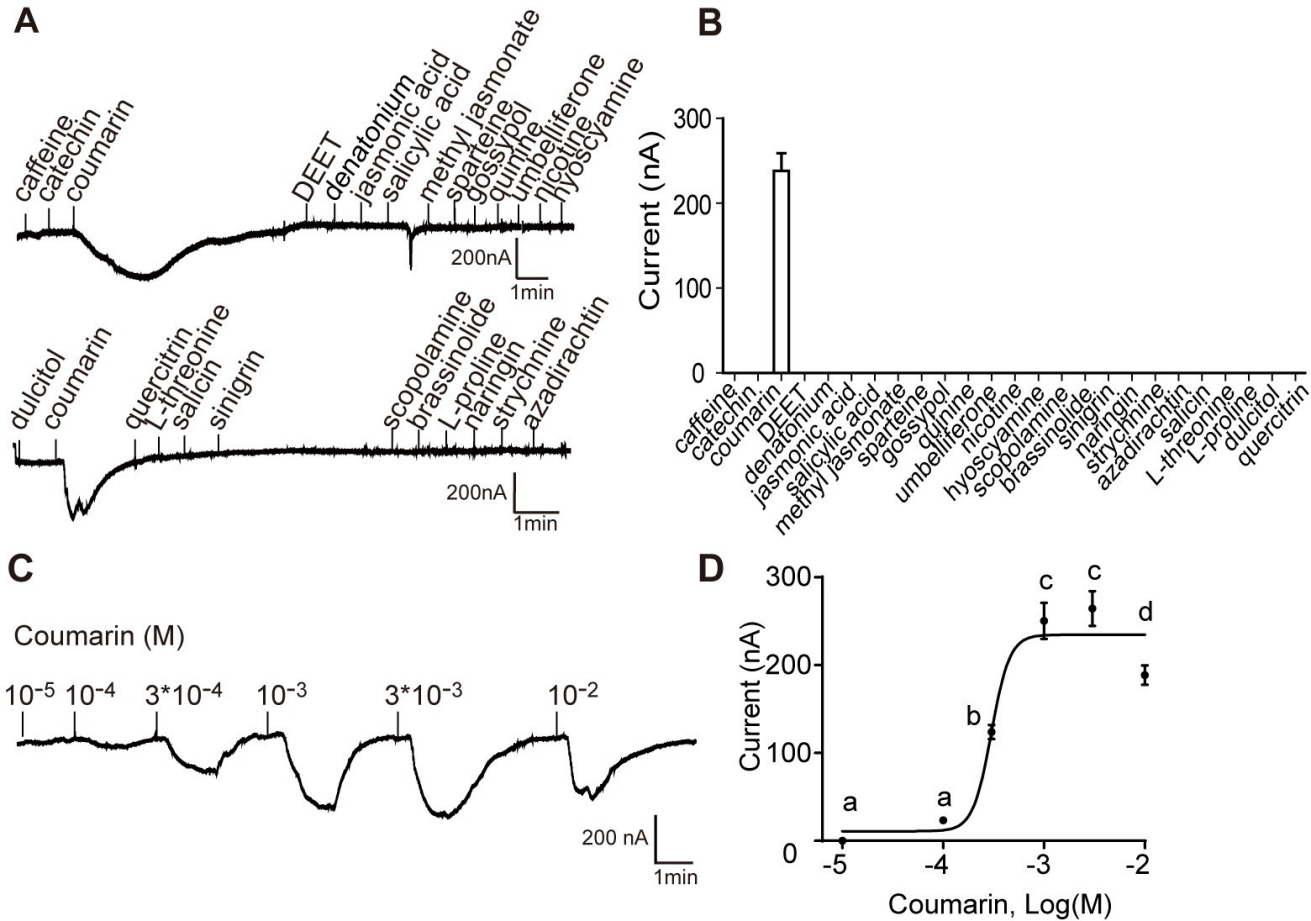
Functional analysis of *Helicoverpa armigera Gr180* in *Xenopus* oocytes. (**A**) Representative inward current responses of *Xenopus* oocytes expressing Gr180 in response to compounds. (**B**) Response profiles of *Xenopus* oocytes expressing Gr180 in response to compounds (n = 8–14). (**C**) Representative inward current responses of *Xenopus* oocytes expressing Gr180 in response to coumarin at a range of concentrations. (**D**) Dose responses of *Xenopus* oocytes expressing Gr180 to coumarin (n = 8). Data are mean ± SEM. Different letters are significantly different at p < 0.05 (one-way ANOVA followed by post-hoc analysis with Tukey’s HSD test).

Oocytes expressing Gr180 specifically responded to 10^−2^ M coumarin, with an average current of 241 nA but did not respond to other tested chemicals (Fig 2A and 2B). Oocytes expressing Gr180 also showed dose-dependent responses to coumarin from the lowest threshold concentration of 3 × 10^−4^ M (Fig 2C and 2D). As negative controls, oocytes injected with ddH_2_O did not show any response to coumarin (S2 Fig).

We also functionally characterized HarmGr67 and HarmGr68 using the same methods. The amino acid sequences of the two receptor genes shared relatively high similarity with BmGr53 (50% and 45.37%, respectively, S1 Fig), a receptor responding to coumarin and caffeine in *B*. *mori* [31]. The results showed that oocytes expressing HarmGr67 or HarmGr68 did not respond to coumarin or other tested compounds (S2 Fig).

### Coumarin induced the responses of the larval medial sensilla styloconica and inhibited the responses of lateral sensilla styloconica to sucrose

**Fig 3:**
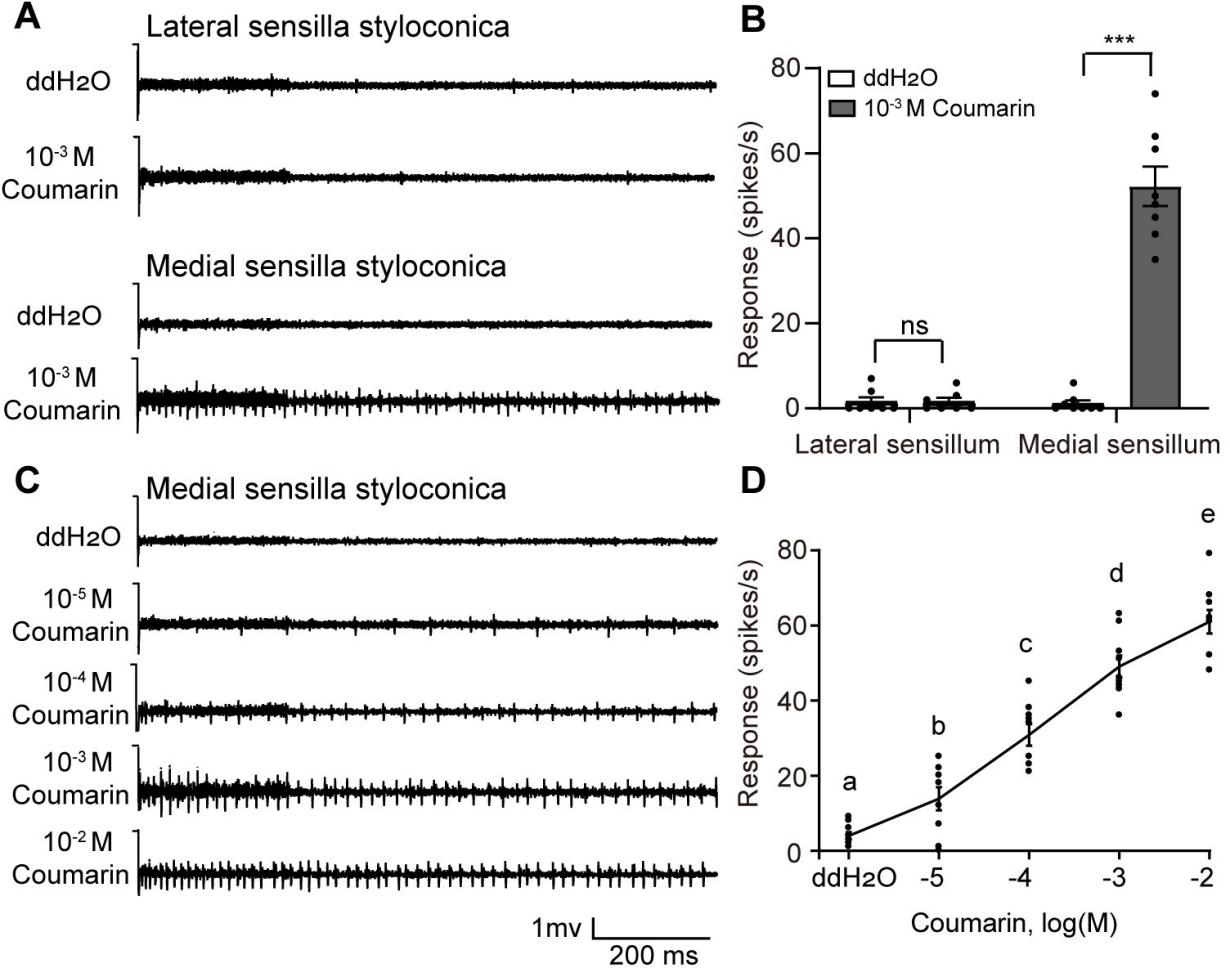
Electrophysiological response of sensilla styloconica on the maxillary galea of *Helicoverpa armigera* larvae to coumarin. (**A**) Representative responses and (**B**) spike frequencies of lateral and medial sensilla styloconica to coumarin and double distilled water (ddH_2_O) at 10^−3^ M (n = 8). *** and ns indicate significant difference (p < 0.001) and no significant difference (p > 0.05), respectively (two-tailed independent samples *t*-test). (**C**) Representative responses of medial sensilla styloconica to coumarin at a series of concentrations. (**D**) Dose responses of medial sensilla styloconica to coumarin (n = 8–10). Different letters indicate significant difference (one-way ANOVA followed by post-hoc analysis with Tukey’s HSD test). Data are mean ± SEM.

To examine whether larvae can perceive coumarin, we tested the electrophysiological responses of the two pairs of sensilla styloconica in the maxillary galea of fifth instar larvae to coumarin using the tip-recording technique. The lateral sensilla styloconica did not respond to coumarin, while the medial sensilla styloconica responded to coumarin at 10^−3^ M (Fig 3A and 3B). The spike frequency of the medial sensilla styloconica induced by coumarin increased from the lowest threshold concentration of 10^−5^ M in a dose-dependent manner (Fig 3C and 3D). Apparently, there is a deterrent cell responding to coumarin in the medial sensilla styloconica of *H*. *armigera* larvae.

To test whether coumarin was able to inhibit sucrose-sensitive cells in the lateral sensilla styloconica, we mixed sucrose and coumarin with different concentrations and examined the response of sucrose-sensitive cells. We found that the spike frequencies of the sucrose-sensitive cells in the lateral sensilla styloconica induced by 10^−2^ M coumarin mixed with 10^−3^ M sucrose or 10^−2^ M sucrose reduced by 46.43% or 41.56% compared with the frequencies induced by 10^−3^ M or 10^−2^ M sucrose alone, respectively. However, 10^−3^ M coumarin did not inhibit the responses of 10^−3^ M sucrose or 10^−2^ M sucrose (S3 Fig). This result indicates that high concentrations of coumarin significantly inhibited the response of sucrose-sensitive cells in the lateral sensilla styloconica. The same method was used to test whether coumarin could inhibit inositol-sensitive cells in the medial sensilla styloconica. The results showed that 10^−2^ M coumarin did not inhibit the response of the medial sensilla styloconica to inositol (S3 Fig).

### Coumarin inhibited feeding behavior of *H*. *armigera* larvae through contact chemoreception

**Fig 4:**
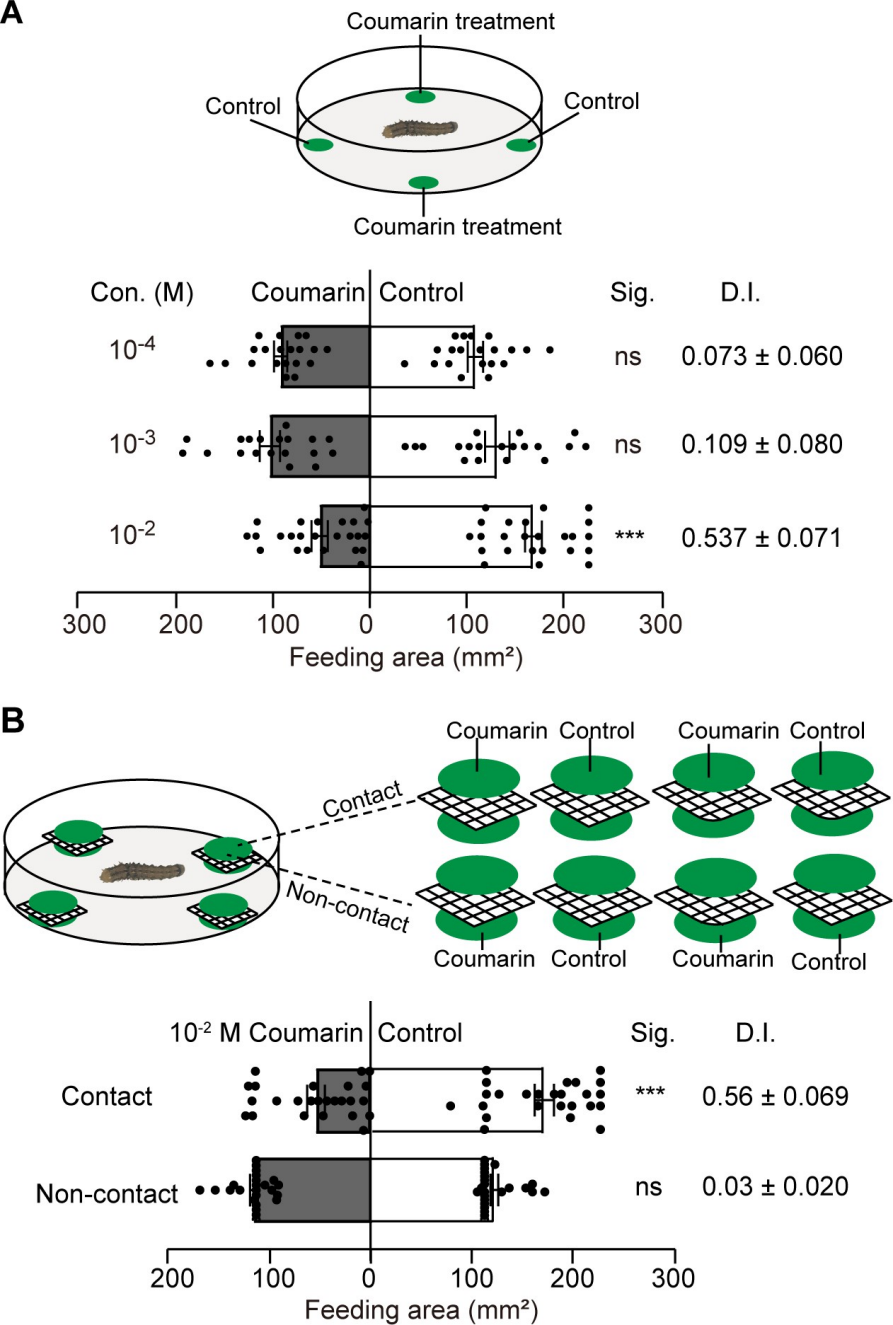
Feeding deterrence of the fifth instar *Helicoverpa armigera* larvae to coumarin by contact chemoreception. (**A**) Two-choice feeding assays with cowpea leaf discs: the feeding area of coumarin-treated discs (grey bars) and control discs (white bars) were measured (n = 19–20). (**B**) Modified two-choice feeding assays with ‘sandwich’ leaf discs: in the contact two-choice feeding assay, coumarin or control discs were painted on the upper leaf discs (n = 25); in the non-contact feeding assay, coumarin or control discs were painted on the lower leaf discs that prevented larvae from feeding (n = 24); the feeding area of the consumed upper leaf discs was measured. Feeding deterrence index (D.I.) = (consumed areas of the control discs—consumed areas of the treated discs) / (consumed areas of the control discs + consumed areas of the treated discs). Data are mean ± SEM. *** and ns indicate significant difference (p < 0.001) and no significant difference (p > 0.05), respectively (two-tailed paired samples t-test).

We tested the effect of coumarin on the feeding behavior of larval *H*. *armigera* on cowpea leaves using two-choice leaf disc assays. The results showed that the feeding areas of larvae were significantly smaller on leaf discs treated with 10^−2^ M coumarin than on the control discs, and the feeding deterrence index was 0.537 ± 0.071. With coumarin at a concentration of 10^−3^ M or 10^−4^ M, the feeding areas of larvae showed no significant difference between the treated and control leaf discs (Fig 4A). Because coumarin is a volatile, we further examined whether contact chemoreception is the key factor for feeding inhibition [38]. The feeding inhibition of coumarin was abolished when larvae were unable to contact leaf discs treated with 10^−2^ M coumarin (Fig 4B), indicating that the inhibitory effect is mediated by contact chemoreception.

### A *Gr180* homozygous mutant strain was established by CRISPR-Cas9

**Fig 5:**
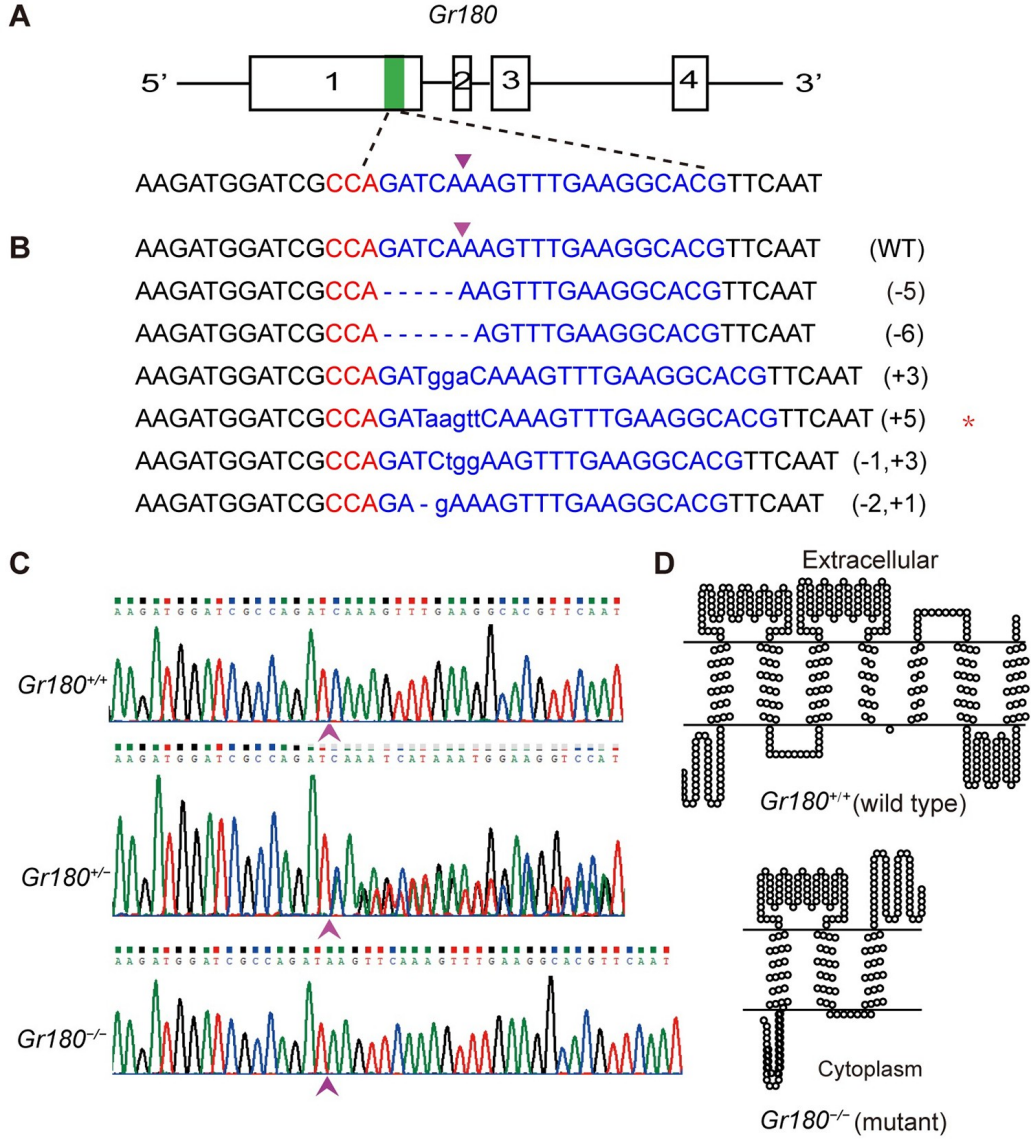
Establishment of *Gr180* homozygous mutants of *Helicoverpa armigera* via CRISPR-Cas9. **(A)** Genomic structure of *Gr180* and designation of sgRNA. Exons are shown as boxes and the lines between two exons indicate the introns. The sgRNA are located on the antisense strand of exon-1 (green box). The sgRNA targeting sequence is shown in blue and the PAM sequence is shown in red. (**B**) Various mutant genotypes of *Gr180* identified by sequencing of the G1 adult PCR products. Purple inverted triangle indicates the cleavage site of Cas9 nuclease. Dashes indicate the deleted bases; lowercase letters are the inserted bases. The numbers of inserted or deleted bases are displayed at the right of each allele (+ insertion;–deletion). Red asterisks indicate the selected genotype to establish the homozygous mutant strain. (**C**) Representative chromatograms of direct sequencing of the PCR products obtained from wild types (*Gr180*^*+/+*^, upper graph), heterozygous mutants (*Gr180*^*+/−*^, middle graph), and homozygous mutants (*Gr180*^*−/−*^, lower graph). The start site of overlapping peaks is marked by a purple arrow. (**D**) Secondary structure prediction of wild type and truncated Gr180 protein. TOPCONS (topcons.net) models were used to predict secondary structure, and TOPO2 software (http://www.sacs.ucsf.edu/TOPO2/) was used to construct the images. In WT, the Gr180 protein consists of seven transmembrane domains, the truncated protein consists of three transmembrane domains in the mutants.

To validate the function of *Gr180 in vivo*, we constructed homozygous mutant strains with truncated Gr180 proteins using the CRISPR-Cas9 system. We designed sgRNAs on the first exon of this gene and obtained a total of six different mutant genotypes (Fig 5A and 5B). Of these, one with a 5-bp insertion was selected to establish the *Gr180* homozygous mutant strain (*Gr180*^*−/−*^) for the largest number of G1 individuals (Fig 5C). This insertion introduced a stop codon and translated into a protein comprising only 239 amino acids, which was 172 amino acids shorter than the wild type Gr180 protein. As the truncated Gr180 proteins contained only three transmembrane domains, we assumed that the mutants lacked the function of Gr180 (Fig 5D).

To test the possibility of off-target effects, biological parameters of the wild type (WT) and *Gr180*^*−/−*^ were compared. There were no differences in terms of fifth instar larval development time, body weight, pupae weight, eclosion rate, adult lifespan, or increased weight and number of feces per larva between the two strains of fifth instar larvae fed on cowpea leaves for 24 h (S4 Fig), suggesting that no off-target effects occurred in this study.

### Knockout of *Gr180* attenuated the sensitivity of medial sensilla styloconica to coumarin, sinigrin, and strychnine in *H*. *armigera* larvae

**Fig 6:**
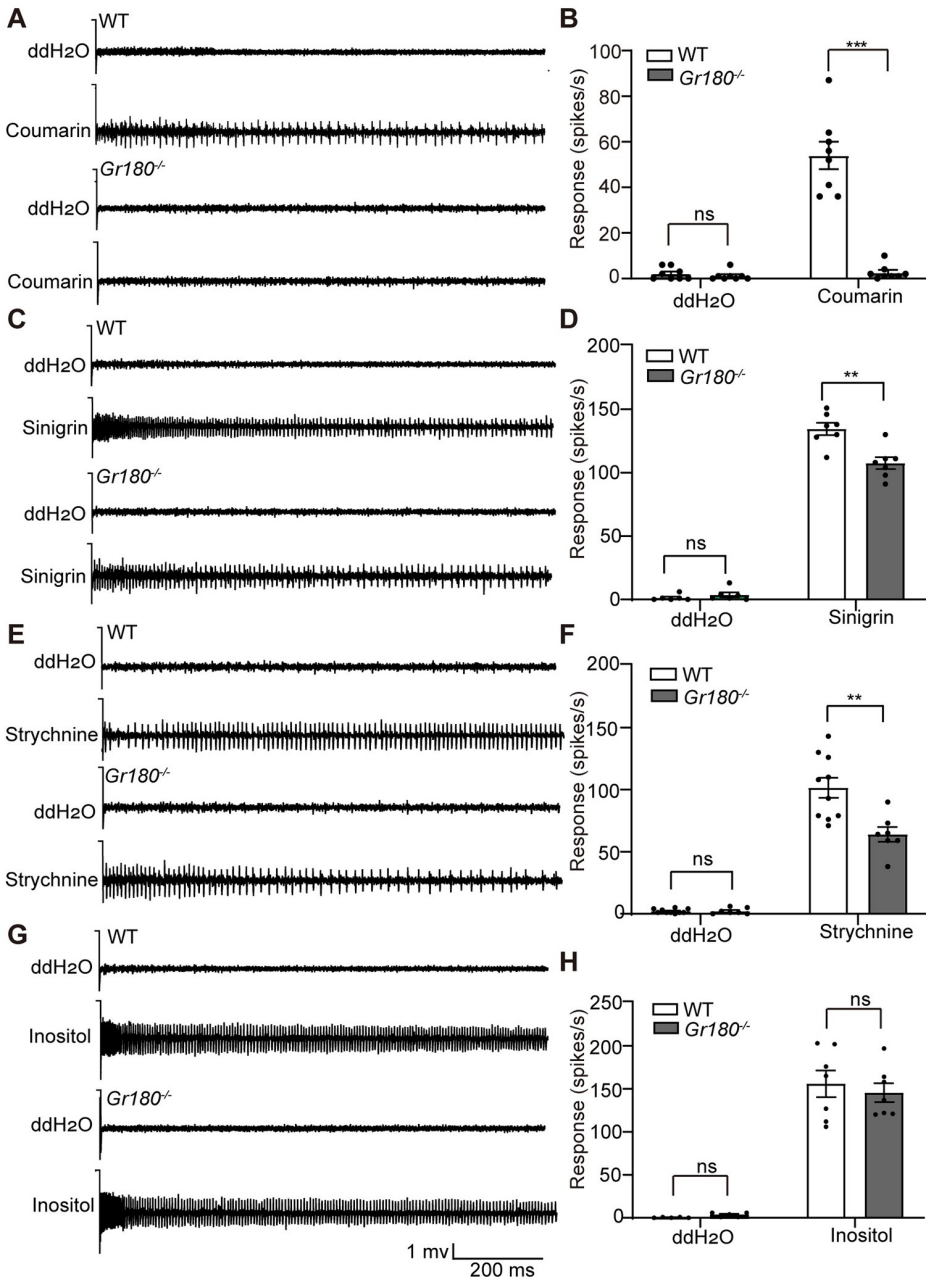
Electrophysiological responses of medial sensilla styloconica in larvae of *Helicoverpa armigera* wild type (WT) and *Gr180*^*−/−*^ mutants. Representative responses (**A**, **C**, **E**, **G**) and spike frequencies (**B**, **D**, **F**, **H**) of medial sensilla styloconica to 10^−2^ M coumarin (A, B), 10^−2^ M sinigrin (**C**, **D**), 10^−2^ M strychnine (**E**, **F**), 10^−3^ M inositol (**G**, **H**). Data are mean ± SEM, n = 7–11. ** and *** indicate significant differences at the level of p<0.01 and p<0.001, respectively; ns indicates no significant difference (p>0.05) (two-tailed independent samples).

The medial sensilla styloconica of *H*. *armigera* larvae are sensitive to coumarin (this study), sinigrin, strychnine, and inositol [34–36]. We compared the electrophysiological responses of the medial sensilla styloconica to these four compounds in *Gr180*^*−/−*^ and WT larvae. The spike frequency of the medial sensilla styloconica in *Gr180*^*−/−*^ larvae induced by 10^−2^ M coumarin was almost abolished, and the spike frequency induced by 10^−3^ M and 10^−2^ M sinigrin or 10^−2^ M strychnine was notably decreased (Fig 6A–6F). However, the spike frequency of the medial sensilla styloconica of *Gr180*^*−/−*^ larvae induced by 10^−3^ M inositol was the same as that in the WT (Fig 6G and 6H). These results indicate that knockout of *Gr180* resulted in a loss of sensitivity to coumarin and significantly reduced sensitivity to sinigrin and strychnine in the medial sensilla styloconica.

The lateral sensilla styloconica of *H*. *armigera* larvae responded electrophysiologically to sucrose and azadirachtin [36]. To test the effects of knockout of *Gr180* on the electrophysiological responses of the lateral sensilla styloconica, the spike frequencies of these sensilla induced by 10^−2^ M sucrose, 10^−3^ M azadirachtin, and the mixture of 10^−2^ M coumarin and 10^−2^ M sucrose were compared between *Gr180*^*−/−*^ and WT larvae. The results showed that the responses of the lateral sensilla styloconica to three tested chemical stimuli showed no significant differences between *Gr180*^*−/−*^ and WT (S5 Fig). These results indicate that the knockdown of *Gr180* has no effect on the responses of the lateral sensilla styloconica induced by sucrose, azadirachtin, and inhibition of coumarin on sucrose neuron.

### Knockout of *Gr180* attenuated feeding deterrent effects of coumarin, sinigrin, and strychnine in *H*. *armigera* larvae

**Fig 7:**
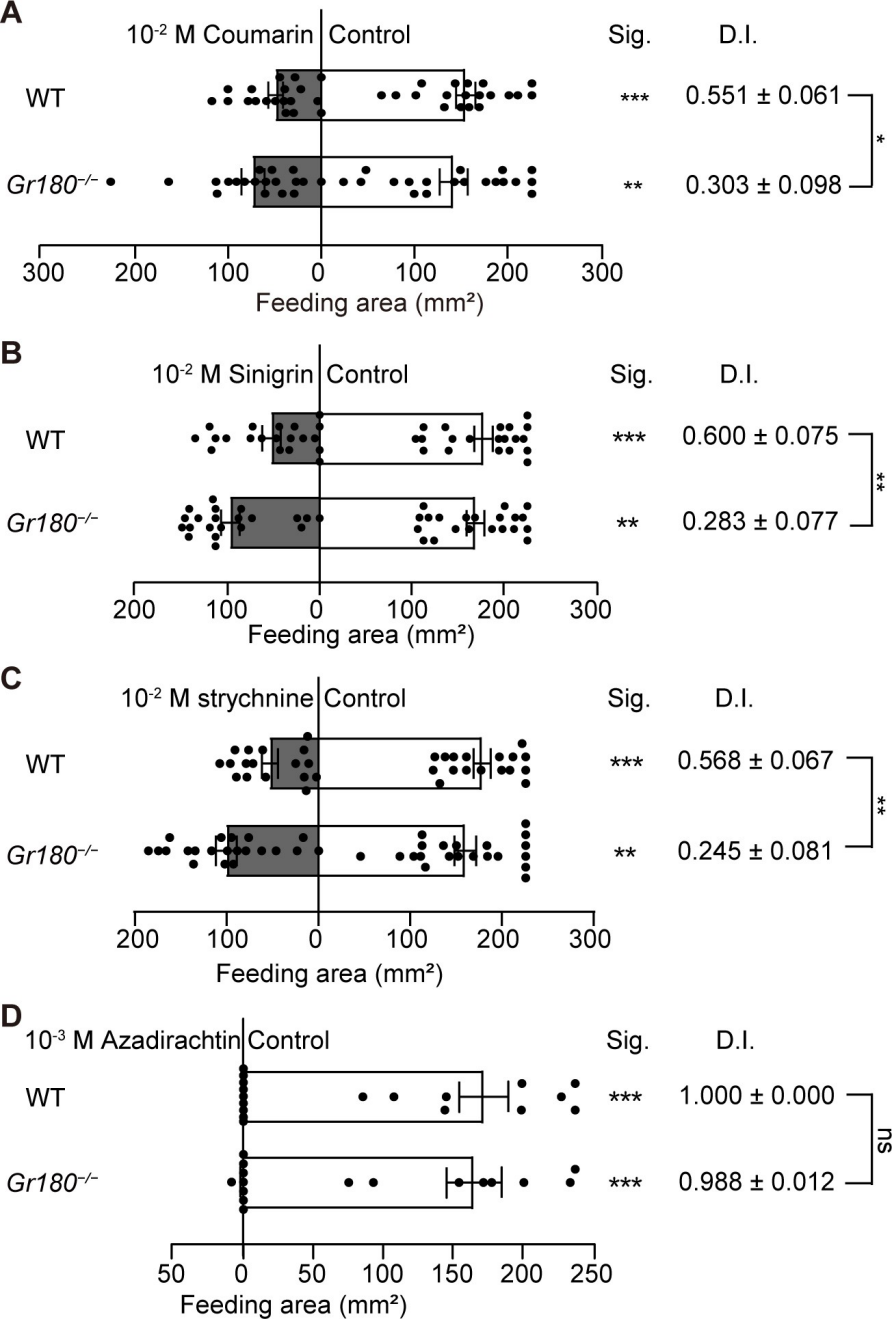
Feeding responses of *Helicoverpa armigera* wild type (WT) and *Gr180*^*−/−*^ mutant larvae to four deterrent compounds. **(A)** Control vs 10^−2^ M coumarin (WT: n = 19; *Gr180*^*−/−*^: n = 19). **(B)** Control vs 10^−2^ M sinigrin (WT: n = 20; *Gr180*^*−/−*^: n = 21). **(C)** Control vs 10^−2^ M strychnine (WT: n = 17; *Gr180*^*−/−*^: n = 21). **(D)** Control vs 10^−3^ M azadirachtin (WT: n = 9; *Gr180*^*−/−*^: n = 8). Data are mean ± SEM. ** and *** indicate significant difference at the level of p < 0.01 and p < 0.001, respectively; ns indicates no significant difference (p > 0.05).

We used two-choice feeding assays to examine the effects of knockout of *Gr180* on the feeding activity of *H*. *armigera* larvae in response to coumarin, sinigrin, strychnine, and azadirachtin. The results showed that coumarin, sinigrin, and strychnine at 10^−2^ M all had a significant feeding deterrent effect on WT larvae, but at 10^−3^ M had no such effect (Figs 4A and S6). The feeding deterrence index of these compounds for WT and *Gr180*^*−/−*^ larvae was compared at a concentration of 10^−2^ M. Coumarin, sinigrin, and strychnine at 10^−2^ M still induced a feeding deterrent effect in *Gr180*^*−/−*^ larvae but the feeding deterrence index was much lower than that of the WT larvae (Fig 7A–7C). The larvae of WT and *Gr180*^*−/−*^ barely fed on leaf discs treated with 10^−3^ M azadirachtin, and there was no significant difference in the feeding deterrence index between WT and *Gr180*^*−/−*^ (Fig 7D). These results indicate that the knockout of *Gr180* attenuates the deterrent effect of coumarin, sinigrin, and strychnine on the feeding of *H*. *armigera* larvae but has no effect on the deterrent effect of azadirachtin.

## Discussion

Taste perception of feeding deterrents is important for host recognition by herbivorous insects but the related molecular sensing mechanisms remain unclear. In this study, we analyzed the function of Gr180, the most highly expressed GR in the larval maxillary galea of *H*. *armigera*, and found that Gr180 was specifically responsive to coumarin. Coumarin was sensed by the medial sensilla styloconica, which resulted in a feeding deterrent effect on *H*. *armigera* larvae. The knockout of *Gr180* using CRISPR-Cas9 almost eliminated electrophysiological responses of the medial sensilla styloconica to coumarin, but coumarin still had lower feeding deterrence on the mutant larvae. Meanwhile, the electrophysiological and behavioral responses of the mutant larvae to sinigrin and strychnine were all decreased.

Herbivorous insects detect feeding deterrents in plants and thereby avoid feeding on toxic compounds, which is crucial for insect survival and adaptation to plant defenses [1]. Coumarins are widely distributed in the plant kingdom; approximately 800 coumarins have been identified [39]. They are mainly synthesized in plant fruits, roots, stems, and leaves. Coumarin concentrations vary greatly among plants, ranging from <2 mg/kg in green tea to 7000 mg/kg in cinnamon bark, and up to 87,000 mg/kg in cassia leaf [40]. Coumarin, as the first of the coumarins to be structurally characterized, not only affects insect feeding and oviposition behavior but also inhibits insect growth and survival rates. Coumarin at a concentration of 0.4 × 10^−2^ M or higher clearly inhibited the feeding of silkworm larvae [31] and 10^−2^ M coumarin inhibited oviposition of *Plutella xylostella* [41]. Concentrations of 0.2% and 10^−2^ M coumarin inhibited larval weight gain in *Porthetria dispar* and *Spodoptera littoralis*, respectively [42,43]. Coumarin decreased the birth rate of the cowpea aphid (*Aphis craccivora*) at low concentrations and increased larval mortality at higher concentrations [44]. Coumarin at 1% significantly increased larvae mortality in *Diaphania hyalinata* (Lepidoptera), *Musca domestica* (Diptera), and *Periplaneta americana* (Blattodea), and adult mortality in *Rhyzopertha dominica* (Coleoptera) [45].

Azadirachtin, strychnine, and sinigrin belong to terpenoids, alkaloids, and glucosinolates, respectively. Azadirachtin, found in *Azadirachta indica* (Meliaceae), is an insecticide that inhibits insect feeding, growth, and reproduction [46]. Lepidopteran larvae are very sensitive to azadirachtin, which had an effective antifeedant effect on many oligophagous and polyphagous insects at concentrations from 10^−5^ M to 5 × 10^−4^ M [46]. Strychnine is a typical bitter substance, first identified from the genus *Strychnos* in Loganiaceae. Strychnine at 2 ×10^−5^ M and 10^−5^ M inhibited feeding of the specialist insects *B*. *mori* and *Pieris brassicae*, respectively [8,47]. At 0.5 × 10^−2^ M and 10^−2^ M, strychnine inhibited the feeding of the generalist insects *Lymantria dispar* and *Mamestra brassicae*, respectively [48,49]. Sinigrin, a glucosinolate found in certain plants of Brassicaceae, inhibits feeding of many insects, but stimulates feeding of some specialist insects on mustard plants. Sinigrin at concentrations of 10^−3^ M to 10^−2^ M deterred larval feeding of the polyphagous insects *M*. *brassicae* and *M*. *configurata* [49,50]. Sinigrin at a concentration of 3 × 10^−5^ M deterred feeding of the oligophagous insect *Papilio polyxenes* [51]. However, 10^−5^ M and 10^−6^ M sinigrin had a stimulatory feeding effect on *P*. *rapae* and *P*. *xylostella*, respectively [33,52]. The deterrent cells in taste sensilla of oligophagous insects are more sensitive than those of polyphagous insects, which partially accounts for the different concentration thresholds of plant secondary substances for inhibiting feeding [36,53]. In this study, we found that coumarin, strychnine, and sinigrin significantly inhibited feeding of *H*. *armigera* larvae at high concentrations (10^−2^ M). It seems that polyphagous insects are more tolerant to these bitter compounds.

Deterrent cells are present in two pairs of sensilla styloconica on the maxillary galea of lepidopteran larvae [10,34,49,54,55]. A deterrent cell in the medial sensilla styloconica of *B*. *mori* was sensitive to a number of plant alkaloids and phenolics, including coumarin and strychnine [7,8]. Both lateral and medial sensilla styloconica of *M*. *brassicae* are sensitive to strychnine and sinigrin, and the responses of the latter are significantly stronger than those of the former [49]. The lateral sensilla styloconica of *S*. *littoralis* and the medial sensilla styloconica of *Heliothis virescens*, *Helicoverpa assulta*, and *H*. *armigera* responded to sinigrin [34,55]. The medial sensilla styloconica of *P*. *brassicae* exhibited vigorous responses to strychnine and azadirachtin [9,49]. In *H*. *armigera*, a deterrent cell of the medial sensilla styloconica responded to sinigrin and strychnine, and a deterrent cell of the lateral sensilla styloconica responded to azadirachtin [34–36]. In this study, we confirmed these results in *H*. *armigera*, and found that the deterrent cells of the medial sensilla styloconica are also sensitive to coumarin. It appears that the deterrent cells in the medial sensillum styloconicum of *H*. *armigera* have a broad spectrum, suggesting that multiple bitter GRs are expressed or a universal sensing mechanism is at work.

Many bitter GRs of lepidopteran insects have been identified but only a few have been functionally characterized. PxutGr1, a bitter receptor expressed in the forelegs of *P*. *xuthus*, is tuned to synephrine, which is an oviposition stimulant [30]. The bitter receptors BmGr16 and BmGr53 of *B*. *mori* are tuned to coumarin and caffeine, respectively, and BmGr18 is responsive to coumarin, caffeine, and pilocarpine [31]. PxylGr34, expressed in the medial sensilla styloconica of *P*. *xylostella*, is tuned to the plant hormones brassinolide and 24-epibrassinolide and inhibits larval feeding and female oviposition [32]. PrapGr28, a bitter receptor expressed in the lateral sensilla styloconica of larvae and the medial sensilla of adult legs in *Pieris rapae*, is tuned to sinigrin and stimulates larval feeding and female oviposition [33]. These studies indicate that a single bitter GR may be responsive to one or several bitter compounds. Further studies are needed to determine whether these receptors may also function in combination with other bitter GRs to sense other secondary plant metabolites. The knockout of *BmGr66*, a bitter receptor in *B*. *mori*, using CRISPR-Cas9 resulted in expansion of the feeding range of silkworm larvae but its ligand is still unknown [56]. In this study, we found that the *Xenopus* oocytes expressing Gr180 specifically responded to coumarin. When *Gr180* was knocked out in *H*. *armigera*, the deterrent cells in the medial sensilla styloconica lost their responses to coumarin and become less responsive to sinigrin and strychnine; the feeding deterrence indexes of these three compounds were also reduced. It is worth noting that coumarin still had lower feeding deterrence for the mutant larvae. There are two possible reasons. Firstly, coumarin may affect larval feeding response by inhibiting the activity of sugar cells, on which *Gr180* knockout had no effect. Secondly, there may be other GRs sensitive to coumarin in deterrent cells of *H*. *armigera* larvae. It has been showed that BmGr16, BmGr18 and BmGr53 were involved in recognition of coumarin in *B*. *mori* [31]. These results indicate that single Gr180 is responsive to coumarin and is also involved in the perception of sinigrin and strychnine. Additional studies are needed to determine which other bitter receptors are expressed in the deterrent cells of the medial sensilla, and how multiple bitter receptors coordinate and determine the sensory properties of the cells. In larvae with truncated Gr180 proteins, the electrophysiological response of the medial sensilla styloconica to sinigrin and strychnine decreased but was not abolished, implying that there is a parallel mechanism to sense sinigrin and strychnine, which is independent of Gr180.

Previous studies have shown that the perception of bitter compounds in *Drosophila* usually depends on the heteromeric complex(es) formed by multiple bitter GRs [20,57]. The knockout of any one of three bitter receptors, DmGR10a, DmGR32a, and DmGR33a, decreased avoidance behavior and almost abolished the response of the labellum to nicotine [58]. However, these three bitter receptors were misexpressed in the sugar-sensitive GSN of L sensillum and did not induce a response to nicotine [58]. This finding indicates that more than three bitter receptors are needed to be tuned to nicotine in flies. Similarly, five bitter receptors, DmGR66a, DmGR33a, DmGR32a, DmGR22e, and DmGRGr47a, are strychnine receptors but not the full repertoire of GRs for detecting strychnine [23, 59]. *Drosophila* sweet-sensing neurons misexpressed four bitter receptors (DmGR33a, DmGR39a.a, DmGR66a, and DmGR93a) conferred responses to caffeine, umbelliferone, coumarin, theobromine, and theophylline, and a lack of any one of these receptors eliminated the response of sweet-sensing neurons to the above five bitter compounds [20]. In mammals, one bitter receptor is tuned to one or more bitter compounds [60, 61] The human bitter taste receptors hTAS2R10 and hTAS2R14 were both tuned to coumarin [61]. Thus, the sensing mechanisms of bitter receptors across species appear to be diverse. *H*. *armigera* is a typical polyphagous insect and its bitter receptor family has experienced a significant expansion, with 180 bitter GRs identified [26,29]. A full understanding of the taste coding of feeding and oviposition deterrents from plants is critical for elucidating the evolution of the host range of this agricultural pest. Our study paves a way for investigating the function of bitter receptors in this non-model insect species.

Insect food selection is a trade-off between sensing of feeding stimulants and deterrents. Whether insects feed depends on the quality and quantity of stimulants and deterrents in the food and the starvation state of the insect [62]. In addition to the central taste nervous system, informational integration of feeding stimulants and deterrents also occurs at the periphery. Bitter compounds can directly inhibit the activity of sugar cells in taste sensilla [9,36,63–65]. A previous study showed that 10^−6^ M toosendanin markedly inhibited the activity of sucrose-sensitive cells in the lateral sensilla styloconica of *P*. *brassicae* [9]. Tannic acid, gossypol, and tomatine at a concentration of 10^−3^ M significantly inhibited the activity of sucrose-sensitive cells in the lateral sensilla styloconica of *H*. *assulta* [36]. This study also showed that 10^−2^ M coumarin suppressed the activity of sucrose-sensitive cells of the lateral sensilla styloconica in *H*. *armigera*. The molecular mechanisms behind this phenomenon have been investigated in *Drosophila*. The inhibition of the sugar-cell activity by some bitter compounds depended on the odorant binding protein, OBP49a expressed in accessory cells of the gustatory sensilla of *Drosophila* [66]. Other studies showed that sugar and bitter neurons activated GABAergic interneurons, which in turn inhibited the activity of the sugar neurons [67]. Knockout of *Gr180* had no effect on the inhibitory activity of sucrose neurons by coumarin, indicating that Gr180 was not involved in this inhibitory process. The larvae of the *Gr180*^*−/−*^ mutant still showed aversive behavior to coumarin, suggesting that the inhibitory function of coumarin to sucrose-sensitive neurons or other coumarin-responding receptors was still at work.

### Summary

In this study, we functionally analyzed Gr180, a bitter receptor highly expressed in the maxillary galea of *H*. *armigera* larvae using the *Xenopus* oocyte expression system and CRISPR-Cas9. Gr180 is singly responsive to coumarin, a feeding deterrent for *H*. *armigera* larvae, and is also involved in the detection of another two feeding deterrents, sinigrin and strychnine. Our study provides an effective path to investigate the gustatory perception of herbivorous insects to defensive secondary substances in plants.

## Materials and methods

### Ethics statement

Animal experimentation: All the experimental protocols were approved by the Animal Care and Use Committee of the Institute of Zoology, Chinese Academy of Science (Protocol Number IOZ17090-A). *Xenopus laevis* was anesthetized by bathing in ice water in 30 min before surgery. After that, the animals were reared alone to avoid infection. Necessary protocols were strictly followed to minimize suffering.

### Insects and plants

*Helicoverpa armigera* larvae were collected in a tobacco field in the suburb of Luoyang City, Henan Province, China, and were reared in the laboratory of the Institute of Zoology, Chinese Academy of Sciences, Beijing. The insects were kept at 26 ± 1°C with a 16L:8D photoperiod and 55–65% humidity. Larvae were fed with an artificial diet mainly comprising wheat bran, wheat germ, soybean flour, and yeast. Adult moths were fed with 10% honey water. The first-day larvae of the fifth instar were used in all experiments.

The female African clawed frogs (*Xenopus laevis*) were purchased from Haiwei Panshi Biomedical Technology Co., Ltd., Qingdao, China, and reared on pork liver in a laboratory animal house affiliated with the Institute of Genetics and Developmental Biology, Chinese Academy of Sciences, Beijing, China.

Seeds of cowpea *Vigna sinensis* (cultivar: Cui Jiang) were purchased from the Institute of Vegetables and Flowers, Chinese Academy of Agricultural Sciences, Beijing, China. The cowpea plants were grown in a climate chamber at 26 ± 1°C with a 16L:8D photoperiod. Two-to-three-week-old cowpea leaves were used for larval feeding behavior.

### Transcriptome sequencing and expression analysis of GR genes in *H*. *armigera*

Larval maxillary galeae were quickly dissected and immersed into Trizol, and then stored at -80°C for transcriptome sequencing. Three biological replicates of this tissue were collected. Total RNA was isolated using the RNeasy Plus Universal Mini Kit (QIAGEN, Hilden, Germany). The cDNA library was constructed and sequenced on Illumina HiSeq4000 platform (Illumina, San Diego, CA) at Novogene Co., Ltd., Beijing, China. High-quality clean reads were obtained by removing reads with adapter, empty reads, and low-quality reads from raw data. De novo transcriptome was assembled by Trinity v2.4.0 [68]. The *GRs* were annotated by BLASTx searching against the *H*. *armigera* genome [29]. The TPM values of putative GR genes were calculated to indicate gene transcript levels using RSEM v1.2.15 software [69]. The open reading frames (ORFs) were predicted by the ORF finder (https://www.ncbi.nlm.nih.gov/orffinder/).

### RNA isolation and cDNA synthesis

The fifth instar larvae tissues including mouthparts, heads (without mouthparts), thorax, abdomen, and midgut as well as the 2-3-day-old adult tissues including antennae, proboscises, heads (without antennae and proboscis), foreleg tarsi, thorax, abdomen, wings, and ovipositors were quickly dissected and frozen in liquid nitrogen, and then stored at -80° C. Total RNA was extracted according to the manufacturer’s protocol of the RNeasy Plus Universal Mini Kit (QIAGEN, Hilden, Germany). The concentration and purity of RNA were measured with a spectrophotometer (NanoDrop 2000; Thermo Fisher Scientific, Waltham, MA, USA). cDNA templates were synthesized from 1.6 μg total RNA by M-MLV Reverse Transcriptase (Promega, Wisconsin, WI, USA). The cDNA products were stored at -20°C.

### Phylogenetic analysis

To define the orthology between GRs of *H*. *armigera* and *B*. *mori*, the phylogenetic tree was constructed based on published GRs [26,70]. Briefly, amino acid sequences of the GRs were aligned with MAFFT v7.455 [71], and gap sites were removed with trimAl v1.4 [8]. Maximum likelihood phylogenies were inferred using IQ-TREE v1.6.8 under the Jones-Taylor-Thornton (JTT) + F + G4 model for 5000 ultrafast bootstraps [72]. The phylogenetic tree was visualized and graphically edited in FigTree v1.4.4 (http://tree.bio.ed.ac.uk/software/figtree/).

### GRs cloning

Based on the nucleotide sequences of GRs from *H*. *armigera* genome, we designed specific primers. *Gr180* (HaOG200922) F: 5 ’GCAAGTTAGTGATATTATAAAACCTG3’, R: 5 ’TCAATTCACACTTTGTAACAATATTATG3’; *Gr67* (HaOG200634) F: 5 ’ATGGCGA ACGTAAAAAAAGTAGAAC3’, R: 5 ’TCACACAAAATGTGATATTTGAATA3’; *Gr68* (HaOG200632) F: 5 ’ATGGACGATAAGGAACAAGATAATG3’, R: 5 ’TTAGGAAATGCGAAATATGATA3’. GR amplification from antennae was performed using Q5 High-Fidelity DNA Polymerase (New England Biolabs, Beverly, MA, USA). The PCR condition was as follows: 98°C for 30 s, followed by 35 cycles of 98°C for 10 s, 56°C for 30 s, and 72°C for 40 s, with a final extension at 72°C for 2 min. Finally, the sequence of three bitter GRs was checked by Sanger sequencing.

### Quantitative real-time PCR (qRT-PCR)

*Gr180* specific primers were designed by Primer-BLAST (http://www.ncbi.nlm.nih.gov/tools/primer-blast/), F: 5 ’ACCTCTTGCTAACGGAACAAGT3’, R: 5 ’TCGCTGTGACCCGACAATAA3’. qRT-PCR was performed with SYBR Premix Ex Taq II (Tli RNaseH Plus; TaKaRa, Shiga, Japan) on a QuantStudio 3 Real-Time PCR System (Thermo Fisher Scientific, Waltham, MA, USA). Ribosomal protein S15 (*RPS15*, GenBank number: AY818611.1) was used as the reference gene [73]. Three biological replicates were run for each tissue. The relative expression levels of *Gr180* were calculated according to the 2^−ΔΔCT^ method [74].

### Functional analysis of bitter HarmGRs expressing in *Xenopus laevis* oocytes

The protocol of ectopic expression of *Gr180*, *Gr67*, and *Gr68* in *X*. *laevis* oocytes and the two-electrode voltage clamping was as described previously [75]. Whole-cell currents of the oocytes responding to 25 phytochemicals were recorded by two-electrode voltage clamping. The concentration of each phytochemical was prepared based on their solubility in Ringer solution: coumarin, 10^−2^ M; umbelliferone, 10^−2^ M; dulcitol, 10^−2^ M; N-Diethyl-m-toluamide, 10^−2^ M; L-threonine, 10^−2^ M; L-proline, 10^−2^ M; caffeine, 10^−2^ M; (+/-) catechin hydrate, 10^−3^ M; denatonium benzoate, 10^−3^ M; (+/-)-jasmonic acid, 10^−3^ M; salicylic acid, 10^−3^ M; methyl jasmonate, 10^−3^ M; quinine, 10^−3^ M; (+/-)-nicotine, 10^−3^ M (higher concentration induced response of oocytes injected with H_2_O); sinigrin, 10^−3^ M; (-/-)salicin, 10^−3^ M; naringin, 10^−3^ M; sparteine, 10^−3^ M; gossypol, 10^−3^ M; hyoscyamine, 10^−3^ M; scopolamine, 10^−3^ M; strychnine hydrochloride, 10^−3^ M; brassinolide, 10^−4^ M; quercitrin, 10^−4^ M; azadirachtin, 10^−4^ M. The detailed information and concentrations of each compound were compiled in S2 Table.

The full-length coding sequences of *Gr180*, *Gr67*, and *Gr68* were first cloned into pGEM-T vector (Promega,), and then subcloned into pCS2+ vectors. The recombinant pCS2+ vectors were linearized by the restriction enzyme Not I (TaKaRa, Shiga, Japan). The linearized recombinant pCS2+ plasmid was used to synthesize cRNA *in vitro* using the mMESSAGE mMACHINE SP6 Transcription Kit (Ambion, Austin, TX, USA). Purified cRNAs were re-suspended in RNase-free water and stored at -80°C until use.

For collecting oocytes, *X*. *laevis* was anesthetized by an ice-water bath for 30 min. Oocytes were surgically collected and cultured immediately in a calcium-free washing buffer (82.5 mM NaCl, 2 mM KCl, 1 mM MgCl_2_, 5 mM HEPES, pH = 7.5). Oocytes were treated with 2 mg/mL of collagenase type I dissolved in the washing buffer. Healthy matured oocytes were chosen, and each was microinjected with 27.6 nL of *Gr180* cRNA, *Gr67* cRNA, *Gr68* cRNA, or ddH_2_O. Injected oocytes were incubated in a bath solution (96 mM NaCl, 2 mM KCl, 1 mM MgCl_2_, 1.8 mM CaCl_2_, 5 mM HEPES, pH = 7.5) supplemented with 5% dialyzed horse serum, 50 mg/mL tetracycline, 100 mg/mL streptomycin and 550 mg/mL sodium pyruvate and placed at a 16°C incubator for 4–6 days. All the experimental protocols were approved by the Animal Care and Use Committee of the Institute of Zoology, Chinese Academy of Science (Protocol Number IOZ17090-A).

The whole-cell current of the oocytes was recorded by the two-electrode voltage clamp. The intracellular glass electrodes were filled with 3 M KCl and exhibited 0.2–2.0 MΩ resistance. Signals were amplified with an OC-725C amplifier (Warner Instruments, Hamden, CT, USA) at a holding potential of -80 mV, low-pass filtered at 50 Hz, and digitized at 1 kHz. Oocytes were stimulated by chemicals using a gravity perfusion system. When a compound clearly activated Gr-expressing oocytes, it was used as a diagnostic compound for the following recordings. For each compound, 8–14 cells were recorded. Data were recorded and analyzed using Digidata 1322A and pCLAMP software (RRID: SCR011323) (Axon Instruments Inc, Foster City, CA, USA).

### Electrophysiological responses of sensilla styloconica in the larval maxilla of *H*. *armigera*

The tip recording technique was used to record electrophysiological responses from the lateral and medial sensilla styloconica in the larval maxillary galea of *H*. *armigera*, the protocol was the same as described previously [76]. Larvae were first reared on artificial diets, and at the late stage of the 4th instar, they were fed with green pepper. For recordings, a larva was decapitated, and a spoon-shaped silver wire was gently inserted into the head from the incision to protrude the maxilla. A glass electrode filled with the chemical solution, into which a silver wire was inserted, was fixed on the micromanipulator to contact with the sensilla styloconica on the maxillary galea. The interval between two stimulations was at least 3 min to avoid sensory adaptation. The neural activity was amplified by a preamplifier and was sampled with a computer equipped with a Metrabyte DAS16 A/D conversion board. The amplifier used an AD 515-K (Analog Devices) integrated circuit in the first stage, yielding < 1 pA input bias current, 1015 Ohm and 0.8 pF input impedance. An interface (GO-box) was used for signal conditioning. This involved a second-order band pass filter (-3 dB frequencies: 180 and 1700 Hz) [52]. Digitized traces were analyzed by the SAPID Tool (version 16.0) [77]. The spike frequency was counted from the first second after stimulation using Autospike v. 3.7 software (Syntech, Hilversum, the Netherlands).

All the tested stimuli except for azadirachtin were dissolved in distilled water, and distilled water served as the control. Azadirachtin was dissolved in 1% ethanol, and 1% ethanol served as the control [9]. The tested stimuli include 10^−5^ M, 10^−4^ M, 10^−3^ M, and 10^−2^ M of coumarin, 10^−2^ M of strychnine, 10^−3^ and 10^−2^ M of sinigrin, 10^−3^ M of azadirachtin, 10^−3^ M and 10^−2^ M of sucrose, the mixture of 10^−3^ M sucrose and 10^−3^ M or 10^−2^ M coumarin, and the mixture of 10^−2^ M sucrose and 10^−3^ M or 10^−2^ M coumarin. 7–12 replicates were run for each experiment.

### Two-choice feeding bioassays

Two-choice feeding assays were used to quantify the feeding efficiency of *H*. *armigera* larvae to coumarin, sinigrin, strychnine, and azadirachtin on cowpea leaves. Circular leaf discs of 1.2 cm in diameter were prepared. Briefly, two treated leaf discs and two control leaf discs were alternately placed around the circumference of a 9.0 cm Petri dish and labeled their positions with a mark pen. Each disc was immobilized with a small piece of parafilm during larval feeding. The upper surface of treated discs was painted with the 20 μL solution of coumarin, sinigrin, strychnine, or azadirachtin using a paintbrush, and the control disc was painted with 20 μL of 50% ethanol. Coumarin, sinigrin, strychnine, and azadirachtin were dissolved in 50% ethanol to reach final concentrations of 10^−3^ M or 10^−2^ M for coumarin, 10^−3^ M or 10^−2^ M for strychnine, 10^−3^ M or 10^−2^ M for sinigrin, and 10^−3^ M for azadirachtin. The day 1 fifth-instar larvae were starved for 2.5 hours, then each caterpillar was gently placed in the center of each Petri dish which was covered with wet filter paper to maintain humidity. The areas of leaf discs were observed every half hour after the larvae started feeding. When the total feeding area reached nearly 50% of the whole leaf discs, the caterpillar was removed. Two more Petri dishes with leaf discs but with no caterpillar were arranged in parallel to calculate the area of the intact discs. Finally, the remaining leaf discs and intact discs were scanned using a DR-F120 scanner (Canon, Tokyo, Japan), and the remaining leaf area was calculated with ImageJ software (NIH) [78]. The scanned images were first converted into a black and white picture, and then the pixels of the discs were measured. The remaining area of the disc was counted based on the pixels of the remaining discs and the pixels and the area of the intact disc. Feeding deterrence index (DI): DI = (consumed areas of the control discs—consumed areas of the treated discs) / (consumed areas of the control discs + consumed areas of the treated discs). For each compound, 17–25 larvae were tested.

To test whether contact chemoreception to coumarin is the key factor in larval feeding inhibition, the above two-choice feeding assay was modified. In each location of leaf discs in the Petri dish, a square of hard nylon mesh (holes of 1 mm^2^, area of 4 cm^2^) was placed between two leaf discs to prevent the larvae from reaching the lower discs. The upper (or lower) leaf discs were painted with 10^−2^ M coumarin or 50% ethanol according to the experimental design of Fig 4B. Finally, the feeding areas of the upper leaf discs were calculated. Twenty-four or twenty-five replicates were run for the contact or non-contact feeding assays, respectively.

### Design and synthesis of single guide RNA *in vitro*

The sgRNA target site of *Gr180* was designed on exon 1 using the CRISPR RGEN tool Cas—Designer (http://www.rgenome.net/cas-designer/). The off-target effect of *Gr180* sgRNA (5’GATCAAAGTTTGAAGGCACG3’) was checked through nucleotide blast in GenBank database (https://www.ncbi.nlm.nih.gov/) and CRISPR RGEN tool Cas—OFFinder (http://www.rgenome. Net /cas-offinder/), and no off-target sites were revealed. The sgRNA was synthesized with the gRNA Synthesis Kit (Thermo Fisher Scientific, Pittsburgh, PA), and purified with the gRNA Clean Up Kit (Thermo Fisher Scientific, Pittsburgh, PA). The concentration of sgRNA was measured by NanoDrop 2000 spectrophotometer (Thermo Fisher Scientific, Waltham, MA, USA). Then, sgRNA was diluted to 400ng/μl in RNase-free water and stored at −80°C.

### Embryo microinjection

First, a wet gauze covering a cage served as an ovipositional substance for gravid *H*. *armigera* adults. The newly-laid eggs within one hour were washed off from the gauze with 1% sodium hypochlorite immediately followed by three washes in distilled water. The eggs were aligned and immobilized on a microscope slide with double-sided adhesive tape. Each egg was injected into one nanoliter mixture of sgRNA (200 ng/μL) and Cas9 protein (150 ng/μL, Thermo Fisher Scientific, Shanghai, China) using PLI-100A microinjection system (Warner Instruments, Hamden, Connecticut). The microinjection was finished within one hour. The slide with the injected eggs was placed in a Petri dish to avoid the contamination of wild-type larvae. After one day, flour was sprinkled on the slide to ensure the survival of the larvae hatched from the injected eggs on the double-sided adhesive tape.

### DNA extraction and mutagenesis detection

Adults were anesthetized by CO_2_, then the distal part (approximately 0.5 cm long) of a hind leg of each individual was cut and used for isolation of individual genomic DNA (gDNA). The target DNA sequences were amplified following TransDirect Animal Tissue PCR Kit procedures (TransGen Biotech, Beijing, China). Gene-specific primer pairs (F: 5’GCTACGCTGAAATGAAACGG3’ and R: 5 ’GATAAGCTTGCTCGCAACGG 3’) were used to amplify the *Gr180* target sequences. The mutations were checked by Sanger sequencing.

### Establishment of *Gr180* homozygote mutants

The G0 chimera moths were distinguished by a cluster of multiple sequencing peaks near the PAM site in the sequencing chromatogram. Each G0 chimeric mutant was backcrossed with 2–3 wild-type adults and reared in a plastic cup (5.3 cm in diameter at the bottom, 9.5 cm at the top and 13.3 cm in height). The G1 adults were regarded as heterozygous mutants only when a cluster of overlapping peaks appeared in the *Gr180* target sequence. The indel type of G1 mutants was inferred by the overlapping peaks and determined by direct sequencing. Heterozygous mutants with the same indel type were in-crossed to generate G2 mutant homozygotes. The G2 homozygous mutants were sibling-crossed to expand the homozygotes. The G3 homozygous larvae were used for tip recording and two-choice feeding experiments.

### Off-target defect detection

To test whether the mutagenesis of *Gr180* posed some off-target effects, we randomly chose 20–23 newly hatched larvae from *H*. *armigera Gr180* mutants and wild-type and compared their developmental processes. The time from newly-hatched to the fifth instar larvae, the body weight of the first-day larvae of the fifth instar, pupal weight, and adult lifespan were recorded. The fifth instar larvae of the wild type and *Gr180* mutants were fed on cowpea leaves for 24 hours, and the increased weight and the number of feces were recorded.

### Statistical analysis

Data were analyzed using SPSS 20.0 (IBM Inc., Chicago, IL, USA). The data of two-electrode voltage-clamp recording, electrophysiological dose-response curves, and gene relative expression levels were analyzed with one-way ANOVA and Tukey’s HSD tests with two tails distribution. The data of two-choice feeding experiments were analyzed with two-tailed paired-samples *t*-test. The compared analysis of electrophysiological and feeding deterrence index in mutant and wild-type larvae was performed using two-tailed independent-samples *t*-test. All figures were prepared with Adobe Illustrator CC 2018 (Adobe Systems, San Jose, CA). The raw data of the figures and statistical analyses in this study are provided in S3 Table.

## Supporting information

S1 Fig Phylogenetic tree of GRs in *Helicoverpa armigera* and *Bombyx mori*. Amino acid sequences are based on previously reported GRs. Bootstrap values are based on 5000 replicates. Harm: *H*. *armigera*; Bm: *B*. *mori*.
(TIF)

S2 Fig Responses of *Xenopus* oocytes expressing HarmGr67, HarmGr68, or injected with distilled water to stimulated compounds. No inward current responses of *Xenopus* oocytes injected with **(A)** HarmGr67, **(B)** HarmGr68, or **(C)** distilled water to tested compounds.
(TIF)

S3 Fig Effects of coumarin on the sucrose-induced responses of lateral sensilla styloconica and the inositol-induced responses of medial sensilla styloconica in *Helicoverpa armigera* larvae. **(A)** Representative electrophysiological responses and **(B)** spike frequencies of lateral sensilla styloconica to 10^−3^ M sucrose or mixture of 10^−3^ M sucrose and coumarin at a series of concentrations (n = 8–12). **(C)** Representative electrophysiological responses and **(D)** spike frequencies of lateral sensilla styloconica to 10^−2^ M sucrose or mixture of 10^−2^ M sucrose and 10^−2^ M or 10^−3^ M coumarin (n = 10–11). **(E)** Representative responses and **(F)** spike frequencies of medial sensilla styloconica to 10^−3^ M inositol or mixture of 10^−3^ M inositol and 10^−2^ M coumarin (n = 6). Data are mean ± SEM. Different letters indicate significant difference (one-way ANOVA followed by post-hoc analysis with Tukey’s HSD test).
(TIF)

S4 Fig Comparison of growth and development between *Helicoverpa armigera* wild type (WT) and *Gr180*^*−/−*^ mutants. **(A)** The duration from neonates to the fifth instar larvae (d). **(B)** The larval weight at the beginning of the fifth instar (g). **(C)** Pupal weight (g). **(D)** The lifespan of adults (d). **(E)** The increased weight and **(F)** the number of feces of fifth instar larvae fed on cowpea leaves in 24 h. Data are mean ± SEM, n = 20–23, ns indicates no difference (p > 0.05, two-tailed independent-samples *t*-test).
(TIF)

S5 Fig Electrophysiological responses of sensilla styloconica in larvae of *Helicoverpa armigera* wild type (WT) and *Gr180*^*−/−*^ mutants. **(A)** Representative responses and **(B)** spike frequencies of lateral sensilla styloconica to 10^−3^ M azadirachtin between WT and *Gr180*^*−/−*^ larvae (n = 6). **(C)** Representative responses and **(D)** spike frequencies of lateral sensilla styloconica to 10^−2^ M sucrose and mixture of 10^−2^ M sucrose and 10^−2^ M coumarin among WT and *Gr180*^*−/−*^ larvae (n = 8–9). **(E)** Representative response and **(F)** spike frequencies of medial sensilla styloconica to 10^−3^ M sinigrin among WT and *Gr180*^*−/−*^ mutants larvae (n = 8). Data are mean ± SEM. Two asterisks and ns indicate significant or no difference (p < 0.01 or p > 0.05, two-tailed independent sample *t*-test).
(TIF)

S6 Fig Feeding deterrence of sinigrin and strychnine to the fifth instar larvae of *Helicoverpa armigera*. (**A**) 10^−3^ M and 10^−2^ M sinigrin (n = 19–20). (**B**) 10^−3^ M and 10^−2^ M strychnine (n = 20). Data are mean ± SEM. The data of feeding areas were analyzed by two-tailed paired samples t-test. *** and ns indicate significant difference (p < 0.001) and no significant difference (p > 0.05), respectively.
(TIF)

S1 Table Putative GRs in the larval maxillary galea of *Helicoverpa armigera*. (DOCX)

S2 Table Tested compounds used for the functional analysis of GRs of *Helicoverpa armigera*. (DOCX)

S3 Table Raw data used in the figures and statistical analyses in this study. (XLSX)
