# Supplementary figures and images for: Functional analysis of a bitter gustatory receptor highly expressed in the larval maxillary galea of Helicoverpa armigera
Source: PLoS Genet. 2022 Oct 7;18(10):e1010455. doi: 10.1371/journal.pgen.1010455 (PMC9581421; doi:10.1371/journal.pgen.1010455)

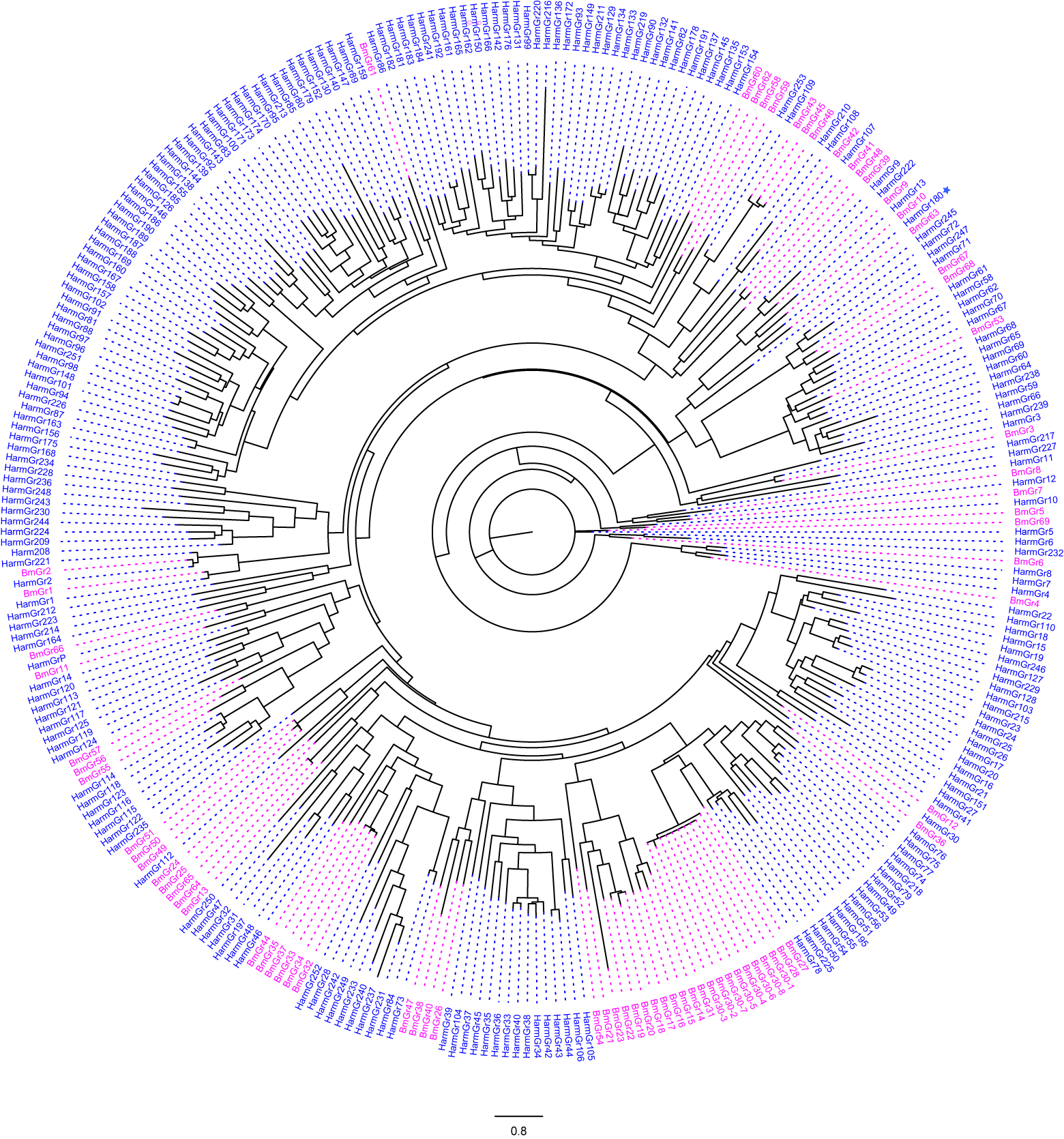

Supplement: S1 Fig — Amino acid sequences are based on previously reported GRs. Bootstrap values are based on 5000 replicates. Harm: H. armigera; Bm: B. mori. (TIF) [file pgen.1010455.s001.tif]

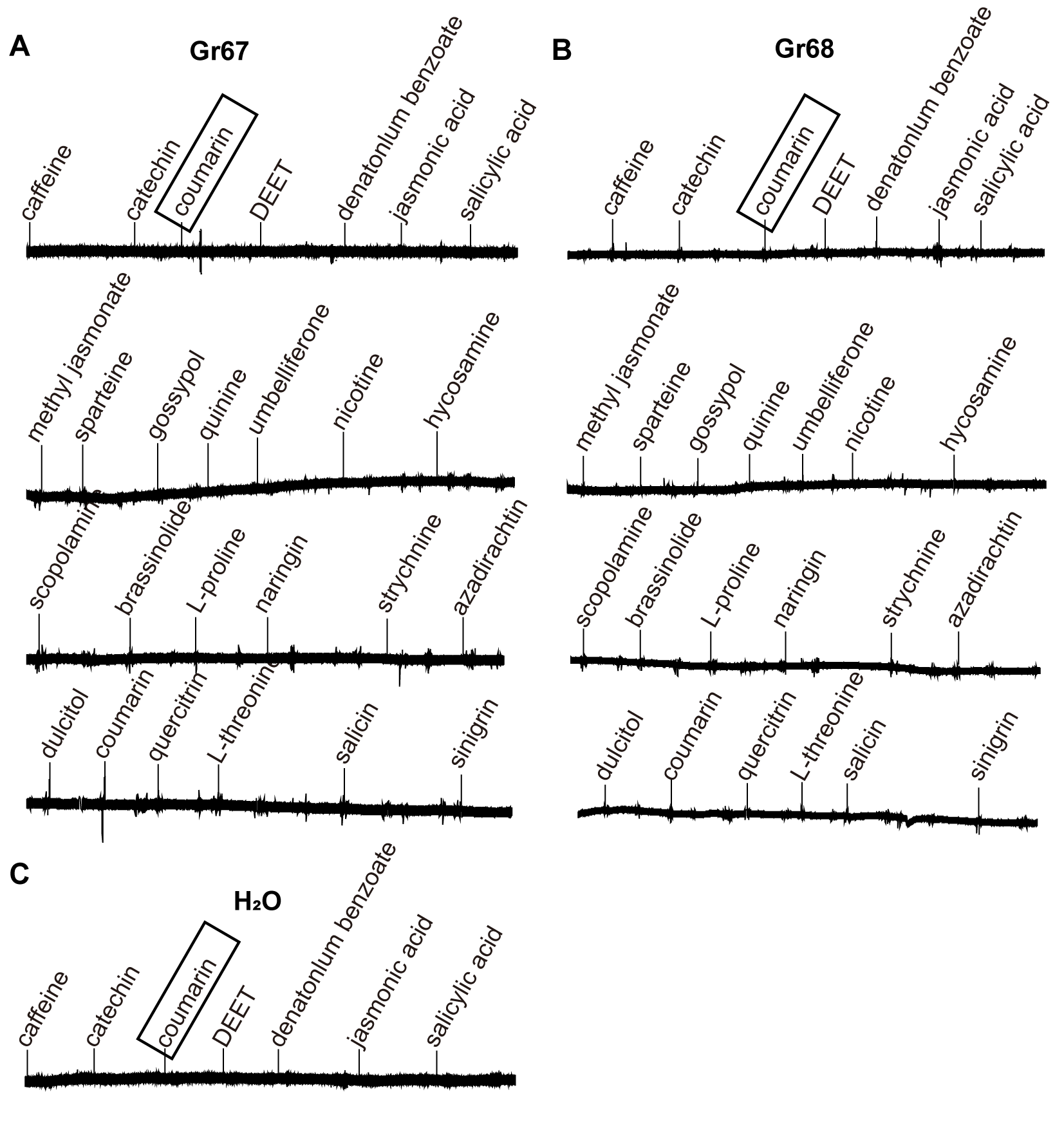

Supplement: S2 Fig — No inward current responses of Xenopus oocytes injected with (A) HarmGr67, (B) HarmGr68, or (C) distilled water to tested compounds. (TIF) [file pgen.1010455.s002.tif]

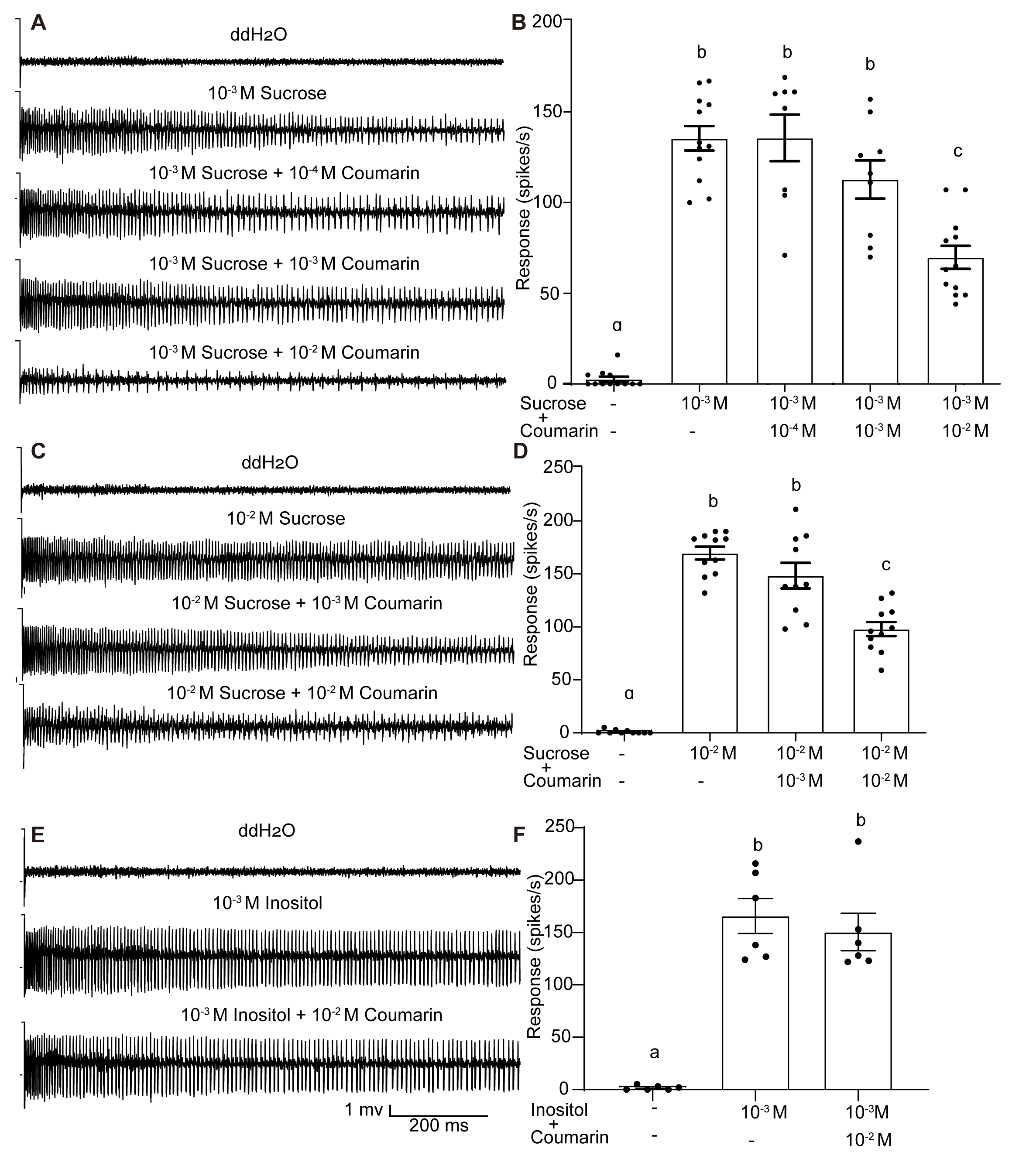

Supplement: S3 Fig — (A) Representative electrophysiological responses and (B) spike frequencies of lateral sensilla styloconica to 10−3 M sucrose or mixture of 10−3 M sucrose and coumarin at a series of concentrations (n = 8–12). (C) Representative electrophysiological responses and (D) spike frequencies of lateral sensilla styloconica to 10−2 M sucrose or mixture of 10−2 M sucrose and 10−2 M or 10−3 M coumarin (n = 10–11). (E) Representative responses and (F) spike frequencies of medial sensilla styloconica to 10−3 M inositol or mixture of 10−3 M inositol and 10−2 M coumarin (n = 6). Data are mean ± SEM. Different letters indicate significant difference (one-way ANOVA followed by post-hoc analysis with Tukey’s HSD test). (TIF) [file pgen.1010455.s003.tif]

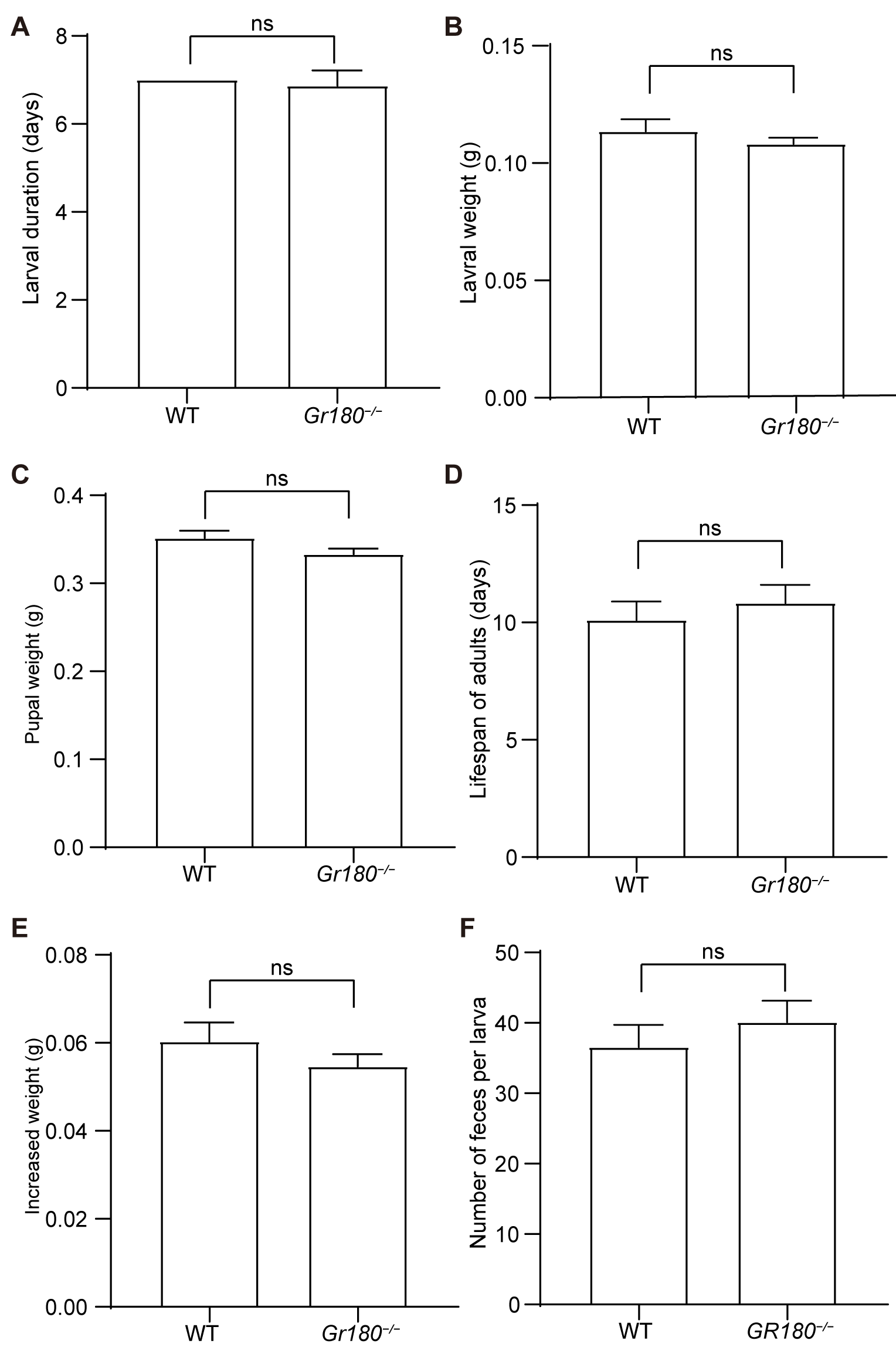

Supplement: S4 Fig — (A) The duration from neonates to the fifth instar larvae (d). (B) The larval weight at the beginning of the fifth instar (g). (C) Pupal weight (g). (D) The lifespan of adults (d). (E) The increased weight and (F) the number of feces of fifth instar larvae fed on cowpea leaves in 24 h. Data are mean ± SEM, n = 20–23, ns indicates no difference (p > 0.05, two-tailed independent-samples t-test). (TIF) [file pgen.1010455.s004.tif]

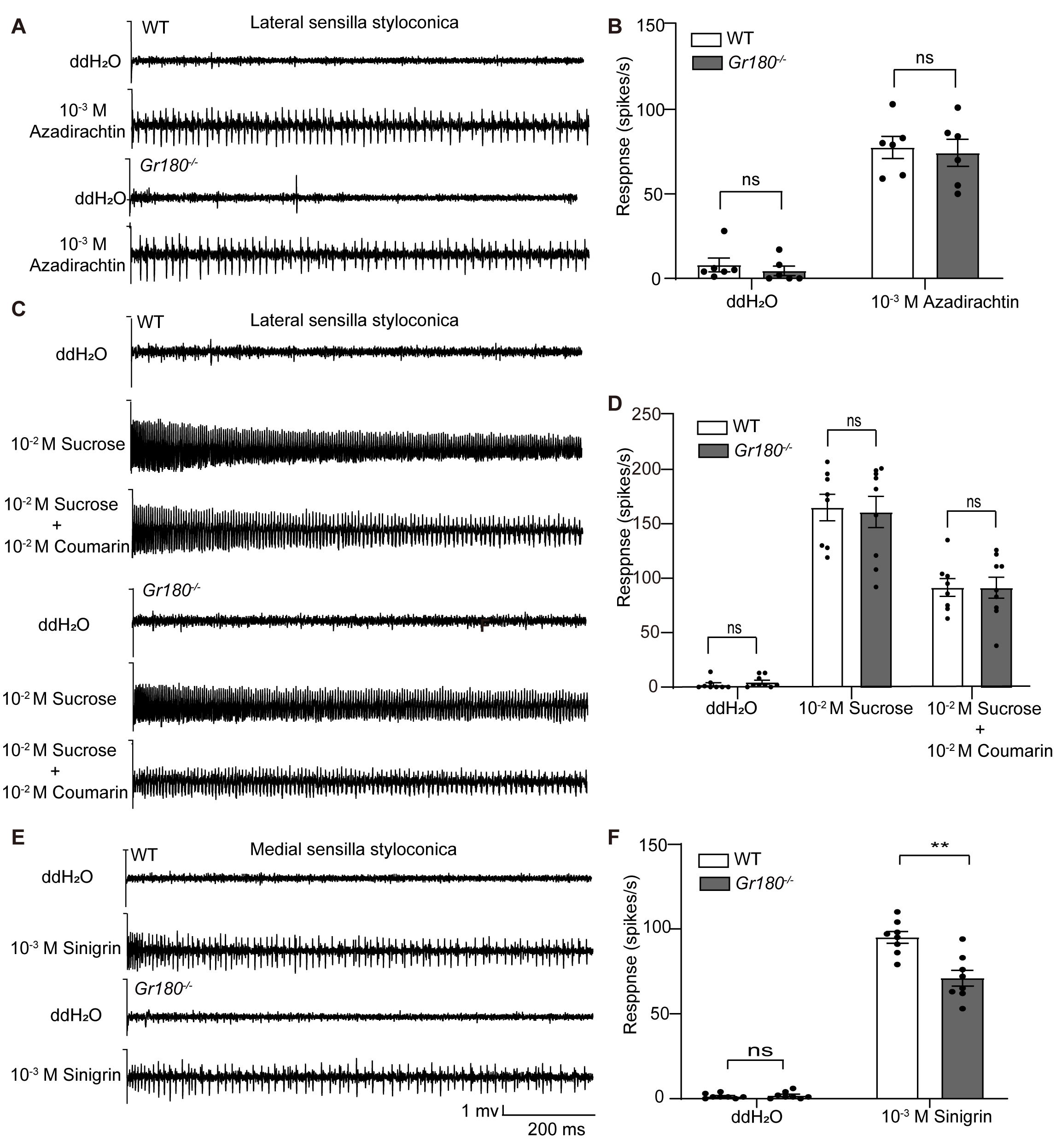

Supplement: S5 Fig — (A) Representative responses and (B) spike frequencies of lateral sensilla styloconica to 10−3 M azadirachtin between WT and Gr180−/− larvae (n = 6). (C) Representative responses and (D) spike frequencies of lateral sensilla styloconica to 10−2 M sucrose and mixture of 10−2 M sucrose and 10−2 M coumarin among WT and Gr180−/− larvae (n = 8–9). (E) Representative response and (F) spike frequencies of medial sensilla styloconica to 10−3 M sinigrin among WT and Gr180−/− mutants larvae (n = 8). Data are mean ± SEM. Two asterisks and ns indicate significant or no difference (p < 0.01 or p > 0.05, two-tailed independent sample t-test). (TIF) [file pgen.1010455.s005.tif]

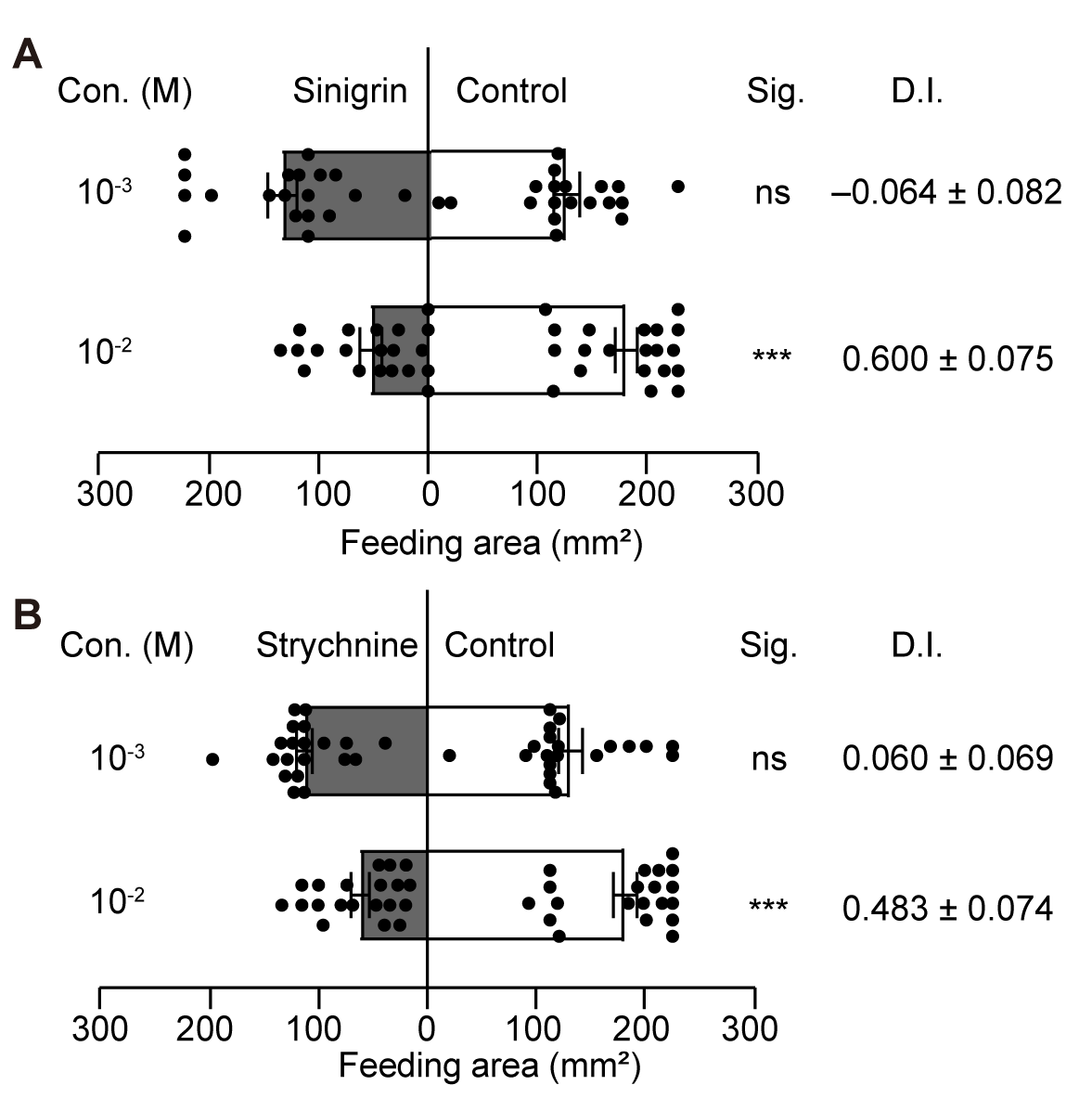

Supplement: S6 Fig — (A) 10−3 M and 10−2 M sinigrin (n = 19–20). (B) 10−3 M and 10−2 M strychnine (n = 20). Data are mean ± SEM. The data of feeding areas were analyzed by two-tailed paired samples t-test. *** and ns indicate significant difference (p < 0.001) and no significant difference (p > 0.05), respectively. (TIF) [file pgen.1010455.s006.tif]
