# Supplementary material for: Functional analysis of a bitter gustatory receptor highly expressed in the larval maxillary galea of Helicoverpa armigera
Source: PLoS Genet. 2022 Oct 7;18(10):e1010455. doi: 10.1371/journal.pgen.1010455 (PMC9581421; doi:10.1371/journal.pgen.1010455)
Supplement: S1 Table — (DOCX) [file pgen.1010455.s007.docx]

**S1 Table. Putative GRs in the larval maxillary galea of *Helicoverpa armigera***

| Gene name | BLAST2  Genome_  Identity(%) | Alignment_Length | E-value | BitScore | TPM(LM_average) | TPM (LM_1) | TPM (LM_2) | TPM (LM_3) |
| --- | --- | --- | --- | --- | --- | --- | --- | --- |
| Gr180 | 98.77 | 405 | 0 | 793 | 1.17 | 1.22 | 1.12 | 1.17 |
| Gr113 | 97.99 | 249 | 2.24E-176 | 500 | 0.85 | 1.00 | 0.74 | 0.82 |
| Gr35 | 94.19 | 86 | 1.08E-39 | 133 | 0.82 | 1.40 | 1.07 | 0.00 |
| Gr136 | 82.76 | 87 | 3.04E-42 | 141 | 0.80 | 2.40 | 0.00 | 0.00 |
| Gr14 | 100.00 | 81 | 1.06E-51 | 169 | 0.70 | 0.00 | 2.09 | 0.00 |
| Gr191 | 95.24 | 105 | 1.06E-68 | 209 | 0.69 | 1.02 | 0.00 | 1.04 |
| Gr119 | 94.12 | 85 | 1.25E-53 | 171 | 0.62 | 1.87 | 0.00 | 0.00 |
| Gr139 | 97.06 | 102 | 2.82E-66 | 202 | 0.58 | 1.14 | 0.00 | 0.59 |
| Gr-fragment | 100.00 | 287 | 0 | 557 | 0.56 | 0.74 | 0.46 | 0.47 |
| Gr183 | 90.74 | 108 | 6.80E-63 | 198 | 0.45 | 1.34 | 0.00 | 0.00 |
| Gr175 | 90.24 | 123 | 8.21E-76 | 230 | 0.40 | 0.00 | 0.66 | 0.54 |
| Gr140 | 82.76 | 87 | 6.25E-43 | 143 | 0.35 | 0.00 | 1.05 | 0.00 |
| Gr66 | 82.17 | 129 | 5.36E-71 | 214 | 0.27 | 0.18 | 0.22 | 0.42 |
| Gr135 | 84.62 | 91 | 5.73E-52 | 168 | 0.25 | 0.00 | 0.00 | 0.76 |
| Gr138 | 97.19 | 249 | 6.73E-164 | 464 | 0.24 | 0.39 | 0.15 | 0.19 |
| Gr148 | 90.91 | 121 | 1.81E-63 | 197 | 0.23 | 0.00 | 0.20 | 0.49 |
| Gr144 | 97.66 | 128 | 1.59E-83 | 251 | 0.22 | 0.42 | 0.11 | 0.13 |
| Gr133 | 100.00 | 126 | 3.84E-75 | 225 | 0.22 | 0.00 | 0.40 | 0.25 |
| Gr173 | 89.39 | 132 | 2.22E-63 | 197 | 0.20 | 0.59 | 0.00 | 0.00 |
| Gr98 | 95.19 | 104 | 4.86E-57 | 179 | 0.18 | 0.00 | 0.00 | 0.55 |
| Gr149 | 86.62 | 157 | 5.70E-94 | 283 | 0.17 | 0.23 | 0.24 | 0.05 |
| Gr10 | 94.98 | 438 | 0 | 755 | 0.17 | 0.17 | 0.12 | 0.22 |
| Gr137 | 96.77 | 186 | 1.88E-128 | 372 | 0.17 | 0.22 | 0.14 | 0.15 |
| Gr68 | 99.76 | 417 | 0 | 849 | 0.17 | 0.20 | 0.16 | 0.14 |
| Gr157 | 93.85 | 260 | 1.32E-149 | 426 | 0.16 | 0.34 | 0.10 | 0.03 |
| Gr61 | 98.65 | 370 | 0 | 675 | 0.15 | 0.23 | 0.12 | 0.11 |
| Gr153 | 91.39 | 151 | 1.62E-93 | 280 | 0.14 | 0.36 | 0.00 | 0.07 |
| Gr126 | 91.49 | 141 | 3.81E-89 | 261 | 0.14 | 0.15 | 0.12 | 0.15 |
| Gr65 | 98.91 | 183 | 7.61E-128 | 368 | 0.13 | 0.20 | 0.00 | 0.19 |
| Gr4 | 98.51 | 402 | 0 | 764 | 0.13 | 0.17 | 0.07 | 0.14 |
| Gr23 | 91.48 | 270 | 9.41E-174 | 496 | 0.12 | 0.16 | 0.12 | 0.09 |
| Gr179 | 87.38 | 103 | 1.88E-62 | 191 | 0.12 | 0.20 | 0.17 | 0.00 |
| Gr6 | 99.54 | 437 | 0 | 869 | 0.12 | 0.19 | 0.08 | 0.09 |
| Gr69 | 98.74 | 238 | 4.15E-171 | 482 | 0.12 | 0.19 | 0.08 | 0.09 |
| Gr88 | 95.08 | 183 | 1.90E-114 | 332 | 0.12 | 0.20 | 0.16 | 0.00 |
| Gr83 | 95.70 | 395 | 0 | 692 | 0.10 | 0.12 | 0.03 | 0.16 |
| Gr13 | 98.68 | 227 | 2.98E-147 | 425 | 0.10 | 0.07 | 0.08 | 0.15 |
| Gr152 | 93.37 | 211 | 1.12E-131 | 374 | 0.10 | 0.15 | 0.00 | 0.15 |
| Gr3 | 94.74 | 380 | 0 | 670 | 0.09 | 0.15 | 0.03 | 0.09 |
| Gr122 | 95.76 | 118 | 1.01E-72 | 224 | 0.09 | 0.27 | 0.00 | 0.00 |
| Gr120 | 97.50 | 120 | 6.06E-68 | 177 | 0.09 | 0.26 | 0.00 | 0.00 |
| Gr174 | 96.97 | 165 | 3.54E-114 | 327 | 0.07 | 0.12 | 0.10 | 0.00 |
| Gr62 | 96.36 | 385 | 0 | 764 | 0.06 | 0.08 | 0.08 | 0.03 |
| Gr99 | 96.09 | 179 | 7.35E-113 | 324 | 0.06 | 0.19 | 0.00 | 0.00 |
| Gr164 | 97.27 | 183 | 1.16E-90 | 271 | 0.06 | 0.08 | 0.07 | 0.04 |
| Gr55 | 89.89 | 89 | 2.05E-50 | 167 | 0.06 | 0.19 | 0.00 | 0.00 |
| Gr2 | 99.31 | 433 | 0 | 834 | 0.06 | 0.04 | 0.02 | 0.12 |
| Gr194 | 94.18 | 103 | 2.98E-53 | 168 | 0.06 | 0.09 | 0.00 | 0.09 |
| Gr1 | 100.00 | 414 | 0 | 863 | 0.05 | 0.07 | 0.04 | 0.05 |
| Gr123 | 90.24 | 164 | 3.19E-92 | 273 | 0.05 | 0.15 | 0.00 | 0.00 |
| Gr114 | 81.88 | 149 | 1.94E-80 | 239 | 0.05 | 0.00 | 0.00 | 0.15 |
| Gr60 | 95.80 | 143 | 2.60E-77 | 238 | 0.05 | 0.00 | 0.14 | 0.00 |
| Gr56 | 95.08 | 122 | 4.15E-73 | 224 | 0.05 | 0.07 | 0.00 | 0.07 |
| Gr168 | 96.09 | 256 | 1.85E-170 | 478 | 0.04 | 0.03 | 0.07 | 0.03 |
| Gr109 | 98.26 | 230 | 2.11E-164 | 462 | 0.04 | 0.07 | 0.00 | 0.06 |
| Gr142 | 93.83 | 162 | 5.07E-90 | 265 | 0.04 | 0.00 | 0.00 | 0.13 |
| Gr86 | 87.84 | 222 | 1.68E-133 | 384 | 0.04 | 0.06 | 0.00 | 0.06 |
| Gr85 | 87.65 | 170 | 7.70E-123 | 282 | 0.03 | 0.06 | 0.00 | 0.03 |
| Gr82 | 95.75 | 259 | 8.82E-168 | 481 | 0.02 | 0.00 | 0.03 | 0.02 |
| Gr198 | 98.96 | 386 | 0 | 715 | 0.01 | 0.00 | 0.00 | 0.04 |
| Gr166 | 98.86 | 262 | 2.26E-173 | 489 | 0.01 | 0.02 | 0.02 | 0.00 |
| Gr182 | 93.68 | 253 | 2.93E-128 | 375 | 0.01 | 0.04 | 0.00 | 0.00 |
| Gr192 | 90.59 | 202 | 2.87E-132 | 375 | 0.01 | 0.00 | 0.00 | 0.04 |

Note: LM: larval maxillary garea
