# Supplementary material for: Functional analysis of a bitter gustatory receptor highly expressed in the larval maxillary galea of Helicoverpa armigera
Source: PLoS Genet. 2022 Oct 7;18(10):e1010455. doi: 10.1371/journal.pgen.1010455 (PMC9581421; doi:10.1371/journal.pgen.1010455)
Supplement: S2 Table — (DOCX) [file pgen.1010455.s008.docx]

**S2 Table.** **Tested compounds used for the functional analysis of GRs of *Helicoverpa armigera***

| **Chemical compound** | **Concentration** | **Souce** | **CAS** | **Class** |
| --- | --- | --- | --- | --- |
| (+/-)Catechin hydrate | 1mM | Sigma-Aldrich | CAS:225937-10-0 | Phenol |
| Coumarin | 10mM | Sigma-Aldrich | CAS: 91-64-5 | Benzopyrone |
| Denatonlum Benzoate | 1mM | Shanghai Aladdin Co., Ltd, China | CAS:3734-33-6 | Synthetic |
| (+/-)-Jasmonic acid | 1mM | Tokyo Chemical Industry Co.,Ltd, Japan | CAS: 6894-38-8 | Plant hormone |
| Salicylic acid | 1mM | Tokyo Chemical Industry Co.,Ltd, Japan | CAS: 69-72-7 | Plant hormone |
| Methyl jasmonate | 1mM | Sigma-Aldrich | CAS: 1211-29-6 | Plant hormone |
| Quinine | 1mM | Dalian Meilun Bio Co., Ltd, China | CAS: 130-95-0 | Alkaloids |
| Umbelliferone | 10mM | Sigma-Aldrich | CAS: 93-35-6 | phenylpropanoid |
| (+/-)-Nictoine | 1mM | Sigma-Aldrich | CAS: 22083-74-5 | Alkaloids |
| Brassinolide | 0.1mM | Yuanyeshengwu Co., Ltd, China | CAS: 72962-43-7 | Plant hormone |
| Sinigrin | 1mM | Sigma-Aldrich | CAS: 3952-98-5 | Glycosides |
| D(-/-)Salicin | 1mM | Sigma-Aldrich | CAS: 138-52-3 | Glycosides |
| Dulcitol | 10mM | Sigma-Aldrich | CAS: 608-66-2 | [Sugar alcohol](https://en.wikipedia.org/wiki/Sugar_alcohol) |
| Naringin | 1mM | Sigma-Aldrich | CAS: 10236-47-2 | Glycosides |
| Quercitrin | 0.1mM | Chengdu Mansite Co., Ltd, China | CAS: 522-12-3 | Flavonoid |
| DEET(N-Diethyl-m-toluamide) | 10mM | Toronto Research Chemicals Co., Ltd, Canada | CAS: 134-62-3 | Synthetic |
| Caffeine | 10mM | Chengdu Push Bio-Technology Co., Ltd | CAS:58-08-2 | Alkaloids |
| Sparteine | 1mM | Shanghai Macklin Biochemical Co., Ltd | CAS:492-08-0 | Alkaloids |
| Gossypol | 1mM | Sigma-Aldrich | CAS: 303-45-7 | Terpenoids |
| Hycosamine | 1mM | Toronto Research Chemicals Co., Ltd, Canada | CAS: 101-31-5 | Alkaloids |
| Scopolamine | 1mM | Toronto Research Chemicals Co., Ltd, Canada | [CAS: 51-34-3](https://www.baidu.com/link?url=EG077TN3Aeo4hoGzl67SpQ4BQyZZtPiOLy-lGdl_0RspZ43J5S2pXCbZs349Eos5zDy8qkFojscbrmZKPYHHbuS-3TI4yH-XN4yxke-jV4_&wd=&eqid=feb86d120000db940000000361a99365) | Alkaloids |
| Strychnine hydrochloride | 1mM | Toronto Research Chemicals Co., Ltd, Canada | CAS: 1421-86-9 | Alkaloids |
| Azadirachtin | 1mM | Toronto Research Chemicals Co., Ltd, Canada | CAS: 11141-17-6 | Terpenoids |
| L-Threonine | 10mM | Sigma-Aldrich | CAS: 72-19-5 | Amino acid |
| L-Proline | 10mM | Sigma-Aldrich | CAS: 147-85-3 | Amino acid |
